# Establishing a health-based recommended occupational exposure limit for isoflurane using experimental animal data: a systematic review protocol

**DOI:** 10.1186/s13643-023-02331-0

**Published:** 2023-09-14

**Authors:** Fréderique Struijs, Carlijn R. Hooijmans, Marije Buijs, Albert Dahan, Sebastian Hoffmann, Romy Kiffen, Daniele Mandrioli, Julia Menon, Merel Ritskes-Hoitinga, Nel Roeleveld, Anne de Ruijter, Gert Jan Scheffer, Vivi Schlünssen, Paul T. J. Scheepers

**Affiliations:** 1 Radboud Institute for Biological and Environmental Sciences, Radboud University, Nijmegen, The Netherlands; 2 Department of Anaesthesiology, Pain and Palliative Care, Radboud University Medical Center, Nijmegen, The Netherlands; 3 Leiden University Medical Center, Leiden, The Netherlands; 4 The Evidence-Based Toxicology Collaboration (EBTC), Johns Hopkins Bloomberg School of Public Health, Baltimore, USA; 5 Cesare Maltoni Cancer Research Center, Ramazzini Institute, Bologna, Italy; 6 Netherlands Heart Institute, Utrecht, The Netherlands; 7 Institute for Risk Assessment Sciences, University Utrecht, Utrecht, The Netherlands; 8 Department for Health Evidence, Radboud University Medical Center, Nijmegen, The Netherlands; 9 Department of Public Health, Danish Ramazzini Centre, Aarhus University, Aarhus, Denmark; 10 National Research Center for the Working Environment, Copenhagen, Denmark

**Keywords:** Inhalation anesthetic, Occupational exposure, Work place standard, Animal studies, Reproductive and developmental toxicity

## Abstract

**Background:**

Isoflurane is used as an inhalation anesthetic in medical, paramedical, and veterinary practice. Epidemiological studies suggest an increased risk of miscarriages and malformations at birth related to maternal exposure to isoflurane and other inhalation anesthetics. However, these studies cannot be used to derive an occupational exposure level (OEL), because exposure was not determined quantitatively and other risk factors such as co-exposures to other inhalation anesthetics and other work-related factors may also have contributed to the observed adverse outcomes. The aim of this systematic review project is to assess all available evidence on the effects of isoflurane in studies of controlled exposures in laboratory animals to derive a health-based recommended OEL.

**Methods:**

A comprehensive search strategy was developed to retrieve all animal studies addressing isoflurane exposure from PubMed, EMBASE, and Web of Science. Title-abstract screening will be performed by machine learning, and full-text screening by one reviewer. Discrepancies will be resolved by discussion. We will include primary research in healthy, sexually mature (non human) vertebrates of single exposure to isoflurane. Studies describing combined exposure and treatments with >  = 1 vol% isoflurane will be excluded. Subsequently, details regarding study identification, study design, animal model, and intervention will be summarized. All relevant exposure characteristics and outcomes will be extracted. The risk of bias will be assessed by two independent reviewers using an adapted version of the SYRCLE’s risk of bias tool and an addition of the OHAT tool. For all outcomes for which dose–response curves can be derived, the benchmark dose (BMD) approach will be used to establish a point of departure for deriving a recommended health-based recommended OEL for 8 h (workshift exposure) and for 15 min (short-term exposure).

**Discussion:**

Included studies should be sufficiently sensitive to detect the adverse health outcomes of interest. Uncertainties in the extrapolation from animals to humans will be addressed using assessment factor. These factors are justified in accordance with current practice in chemical risk assessment. A panel of experts will be involved to reach consensus decisions regarding significant steps in this project, such as determination of the critical effects and how to extrapolate from animals to humans.

**Systematic review registration:**

PROSPERO CRD42022308978

**Supplementary Information:**

The online version contains supplementary material available at 10.1186/s13643-023-02331-0.

## Background

Currently, isoflurane is one of the most commonly used inhalation anesthetics. Isoflurane was introduced for clinical applications in 1979. It maintains cardiac output and cerebral perfusion more effectively than sevoflurane. However, isoflurane, in contrast to sevoflurane, is not suitable as an induction agent because of significant airway irritation and potential complications such as laryngospasm. Recovery is smooth but slower as compared to sevoflurane [1, 2].

In the Netherlands, an estimated 23,000–24,000 workers are occupationally exposed to inhalation anesthetics as part of their work in healthcare, veterinary, and animal research facilities [3]. Worldwide, millions of healthcare professionals are daily working with isoflurane in different jobs, including operating theater staff, recovery room staff, ambulance personnel, midwives, dentistry workers, and workers in veterinary practices and research institutes [4].

In epidemiological studies, occupational exposure to inhalation anaesthetics, including isoflurane, has been associated with several adverse reproductive health outcomes, such as extended time to pregnancy, miscarriage, decreased birth weight, and malformations at birth. In 1997, Boivin conducted a meta-analysis of 19 studies on the prevalence of spontaneous abortions reported by women exposed to inhalation anesthetics (anesthetists, nurse anesthetists, operation theater assistants, dental assistants) [5]. Exposure was assessed by questionnaires. Exposure to inhalation anaesthetics resulted in an overall relative risk (RR) of 1.48 with a 95% confidence interval (95% CI) of 1.40 to 1.58 for self-reported spontaneous abortions. In a subgroup analysis of six high-quality studies, the RR increased to 1.90 (95% CI 1.72–2.09). A limitation of these studies is that other concurrent potential risk factors for spontaneous abortion were not considered [6–8]. Peelen and co-workers (1999) [9] reported on 1686 pregnancies of nurses working in hospitals in The Netherlands. Odds ratios (ORs) ranging from 1.1 to 1.6 were reported for 46 reported miscarriages in nurses with tasks involving inhalation anesthetics, but the 95% CIs included unity. For nurses assisting in the operation theater, an OR of 1.8 (95% CI 1.0–1.4) was observed for pregnancies ending in a miscarriage and persisted after adjustment for covariates, such as heavy lifting, working under time pressure, working in night shifts, and use of chemicals for sterilizing/disinfection, cytostatic drugs, and antibiotics. As scavenging of inhalation anesthetics was not a common practice before 2000 [10, 11], exposures were likely substantially higher in these studies compared to current exposure levels [12].

In 2011, a large registry-based epidemiological study reported on congenital malformations in a cohort of 15,317 singleton live-born children from 9433 mothers working as registered nurses in Canada [13]. Exposure status was assessed by registered information on employment, type of healthcare facility, department, and position, combined with telephone interviews with knowledgeable healthcare personnel. Exposure to anesthetic gases occurred in operation theaters and post-anesthetic recovery rooms. The use of several halogenated anesthetic gases increased over the study period, and isoflurane use was reported by 59–61% of the hospitals for the period 1990–2000. Isoflurane was reported to be associated with congenital anomalies of the eyes (OR = 2.78, 95% CI 1.02–7.59), ear, face, and neck (OR = 3.05, 95% CI 0.94–9.88). The strengths of this study were its large population size and the study design using administrative records, which precludes selection and ascertainment bias as well as recall bias. Limitations were the fact that nurses were exposed to mixtures of inhalation anaesthetics, the small numbers of specific congenital anomalies which were resolved by aggregating anomalies in groups of malformations with potentially different etiology, the lack of individual exposure measurements, the inability to pinpoint the presumed exposure to the sensitive time-window for the occurrence of congenital anomalies, and the lack of information on other occupational exposures and potential confounders. Nevertheless, several clearly increased ORs were observed in this study, while the strengths of the associations increased with the level of exposure.

Regarding the exposure of professional users, our scoping review indicated new exposure and biomarker studies published in the past 30 years. Newton and co-workers [14] studied isoflurane in an experimental setting, involving healthy volunteers in a controlled inhalation exposure to evaluate effects on memory at high concentrations (0.1–0.4 × the minimum alveolar concentration, MAC). Six studies reported on neuropsychological/neurobehavioral endpoints, but no more recent studies were retrieved after the study by Proieti et al. in 2003 [15]. One study reported on general haematological parameters [16]. Some studies observed inconsistent or no effects [17, 18]. In Table S1, a comprehensive overview of results is provided.

All other studies reported on exposures in healthcare facilities. Seven studies did not provide quantitative data on exposure. Some studies described methods for exposure assessment but did not report any exposure levels in the abstract. Another seven studies provided quantified isoflurane exposure levels based on workplace measurements. Studies not showing any quantified data sometimes reported exposure as “low” or “below recommended levels” and in one study above “international recommendations” [19]. In addition to air measurements, Lucchini and co-workers [17] presented levels of different inhalation anesthetics in urine, including isoflurane. Cope and co-workers (2002) presented exhaled breath concentrations as a measure of internal exposure [20]. Other studies reported biomarkers of exposure related to molecular events and cellular responses reflecting “early signals” of subclinical and/or reversible changes including indicators of DNA damage. These studies were published over the past 20 years and typically performed in relatively small populations of less than 10 to several hundreds of study participants in the exposed group and a similar number as internal controls, usually healthcare workers with an unknown level of exposure. In these studies, exposures in workers with direct contact with isoflurane were compared to workers with similar job titles with low or no known exposure to inhalation anaesthetics. Proietti et al. compared hospital staff working with open system to staff working with closed systems [15]. In open systems, an exposure to isoflurane of 11.1 ppm was observed compared to 0.4 ppm for closed systems. Nitrous oxide exposures were reported to be 301 and 4.8 ppm, respectively.

More than 25 studies reported on exposures of inhalation anaesthetics and biomarkers (see Supplementary material 1 for details). The largest group of thirteen studies aimed at the determination of biomarkers for genotoxicity in peripheral blood lymphocytes and exfoliated lymphocytes from buccal smears and reported on different indicators of DNA damage such as micronuclei (MN), sister chromatid exchanges (SCE), chromosome aberrations (CA), and comets. In each of these studies, healthcare workers with exposure to inhalation anesthetics including isoflurane were compared to controls with no known exposure to inhalation anesthetics. One study reported on different glutathione-S-transferase (GST) genotypes related to frequencies of MN and SCE [21]. Two studies reported a wide range of oxidative stress parameters in addition to DNA damage [21–23]. Some of these studies reported on cellular endpoints with relevance to apoptosis such as karyorrhexis and pyknosis. Goto and co-workers (2000) looked at neutrophil apoptosis [24]. Other studies performed blood cell counts (e.g., [25]), and one study reported on pro-inflammatory biomarkers/cytokines [26]. For most biomarkers, changes were observed for exposures in a range of 0.5 to 5 ppm for isoflurane, often in the presence of much higher concentrations of nitrous oxide (12–580 ppm). Most of these changes in exposed groups were reported to differ from biomarker levels observed in controls, even for small numbers of 10–15 workers per group. Only in one study medical residents were followed in time over periods of 8, 16, and 22 months of exposure and compared to controls of similar age with no known exposure [22]. DNA damage was increased with exposure to inhalation anesthetics at all three timepoints, and plasma thiols were observed to be increased at 22 months and glutathione peroxidase both at 16 and 22 months, compared to non-exposed controls.

Based on the available human data consensus evaluations of human data by the Health Council of the Netherlands [27], the DFG MAK Kommission Germany [28] the Swedish OEL Committee [29], and the ACGIH [30] concluded that the available human data on inhalation anaesthetics were not suitable to be used for establishing workplace standards. The reproductive health outcomes reported could not be linked to a specific anesthetic as isoflurane is often combined with other anesthetics. In addition, most studies did not adjust for other known or suspected work-related risk factors, such as physical strains, mental stress, shift work, and chemical co-exposures, e.g., to medical drugs and chemicals used for disinfection. Based on the available evidence in 2002, the Netherlands Health Council therefore concluded: “a lack of appropriate human data precludes assessment of isoflurane for a classification related to fertility, developmental toxicity and effects during lactation” [31]. We expect that animal studies will provide that link between exposure to isoflurane in well-controlled environments to adverse health effects. Depending on the study design, animal experiments usually include a well-defined control group. Therefore, in this proposed project, we will rely on evidence from the available animal studies after critically reviewing those studies. Therefore, the aim of this systematic review project is to assess all available evidence on the effects of isoflurane in studies of controlled exposures in laboratory animals to derive a health-based recommended OEL. Based on a search of the literature, we have verified thtat such systematic review has not been published.

### Feasibility of the review

To explore the availability of studies on isoflurane, we performed a scoping review. The search was done on January 10, 2020 (see Supplementary material 1). A wide range of endpoints was retrieved, all with toxicity parameters relevant to humans based on biomarker studies in worker’s populations (Table S1). We propose a systematic review approach to identify all relevant animal studies and ultimately derive two health-based recommended OELs for workers that are occupationally exposed for 40 h per week (5 days with 8h/day), 48 weeks per year for 40 years, i.e., the reference work period for a working life exposure [32]. Depending on the availability of relevant short-term exposure data, we will also derive an OEL for a short-term exposure level of 15 min to address the situation of peak exposures that are common work-related exposure patterns.

## Methods

This protocol [33] describes the methodology used to search, select, and appraise evidence to support the health-based recommendation for an OEL for isoflurane. The study consists of two components: a systematic review summarizing all adverse effects described in primary research in which healthy vertebrates are exposed to isoflurane compared to a non-exposed control group, and a second component in which an OEL will be derived using the benchmark dose (BMD) approach. The protocol for the systematic review is registered in PROSPERO under no. CRD42022308978.

### Search methods for identification of studies

The search strategy was designed in collaboration with an information specialist of the Radboud University Nijmegen. We performed a systematic search in the following databases (used platform): PubMed (National Library of Medicine), EMBASE (Ovid Elsevier), and Web of Science (Clarivate). The full-search strategy (Supplemental materials 2, 3 and 4) was based on the search components isoflurane and animal. For the animal component of the search, we used a pre-existing search filter [34]. Search results from all databases were combined, and duplicates were removed. The initial search was performed on December 9, 2021. Search strategies and reference lists from reports of health authorities assessing the occupational health risk (or similar) will be used to check the completeness of the search (e.g., [35]).

### Selection of studies

Articles will be selected in two phases: first, by title and abstract screening and second, by full text screening. The title and abstract screening will be conducted using EPPI reviewer. For training the EPPI reviewer algorithm (https://eppi.ioe.ac.uk/https://eppi.ioe.ac.uk/), the first 5000 references will be screened using the Rayyan online screening tool (https://www.rayyan.ai/https://www.rayyan.ai/) by two independent reviewers (MB, FS). Disagreements will be resolved after discussion or with the help of a third researcher. We aim for a sensitivity of 95% (comparable to human screening) and specificity of 80–85%. Full-text screening will be conducted by one reviewer. Disagreements will be discussed and resolved between the two reviewers or discussed with a third reviewer until a consensus is reached.

### Eligibility criteria

Articles will be eligible for this review when they describe primary research in which healthy, sexually mature (non human) vertebrates are exposed to isoflurane in vivo compared to a non exposed control group. The control condition will be “no exposure to isoflurane,” i.e., exposure to defined as atmospheres of filtered and conditioned room air, compressed air, air and oxygen, or nitrogen and oxygen. The intervention will include studies reporting on inhalation of isoflurane exposure with a maintenance concentration below 1% (the concentration at induction may be higher), which is considered the most relevant for workplace exposure. All outcomes, not related to efficacy of isoflurane as inhalation anaesthetic, are of interest and will be documented. Articles will be excluded when they do not adhere to the abovementioned criteria or when animals are suffering from comorbidities, co-interventions took place or when isoflurane exposure is combined with other anaesthetics.

During the title-abstract phase of the review, we focus on part of the abovementioned eligibility criteria, because not all relevant details are reported in the abstract (Table 1).

**Table 1:** Selection of exclusion criteria in screening

**Selection phase**	**Exclusion criterion**	**Remark**
Title—abstract	Not a primary study	-
	Not an in vivo vertebrate (non-human) healthy animal study	-
	Not the aim to study effects of isoflurane (e.g., co-morbidities, co-interventions)	Isoflurane (or any other of the descriptors used for “isoflurane” in the search strategy) are not in the title (see Supplemental materials 2, 3 and 4)
	Obviously very young animals exposed to isoflurane	At this stage, we are unsure whether EPPI reviewer will be able to make a distinction between isoflurane exposure of obviously young animals (which should be excluded) versus maternal exposure and outcomes measured in offspring (which should be included). We are therefore not yet sure if this criterion will be part of the final title abstract screening
	Only studies that reported treatment with 1 vol% isoflurane or higher	-
	No abstract available	-
Full text	Sexually immature animals exposed to isoflurane	Above described criterion is redefined if EPPI reviewer will not be able to interpret sexual maturity per species
	Combined use of multiple anaesthetics	-
	Lowest maintenance dose described in paper higher than 1%	Exposures too high to be relevant in an occupational setting will not be included (Menon et al., 2021)

### Data extraction and management

Two reviewers (FS, AR) will independently extract study characteristics such as study identity, model, and intervention characteristics using specifically designed standard data extraction forms. Any disagreements will be resolved by discussion or if required, by a third reviewer (CH). For all adverse outcomes, we will extract the result of the statistical analysis of each adverse effect (based on the judgement of the manuscript authors). Data will be extracted from text and tables. In case of missing data, the authors will be contacted (with a maximum of two attempts by e-mail).

We will extract the following data:

- Author and year of publication
- Number and type of animals in experimental and control groups (placebo, undisturbed, etc.)
- Species, strain, sex, age, and body weight
- Dose–response data for each endpoint studied, composition of gas mixture, timing and duration of exposure, frequency, type of control, and timing of outcome assessment/duration of follow-up
- All adverse effects including health status of dams to inform maternal toxicity in developmental toxicity endpoints

Finally, we will count and compare the number of positive studies with the number of neutral and negative studies per outcome/adverse effect (e.g., vote counting). Subsequently, for each type of adverse event, we will aim to assess the effect of the presence of pregnancy, exposure time, and exposure concentrations by creating subgroups of studies.

### Dealing with duplicate and companion publications

In the event of duplicate publications, companion documents, or multiple reports of a primary study, we will maximize the yield of information by collating all available data and using the most complete dataset aggregated across all known publications. If required, the author will be contacted for clarification.

### Risk of bias

For all studies that will be selected for OEL calculations, two reviewers (FS, AR) will independently assess the risk of bias. Disagreements will be resolved by consensus or by consultation with a third reviewer (CH). The risk of bias will be assessed using a combination of the SYRCLE’s Risk of Bias tool [36] and OHAT Risk of Bias Rating Tool for Human and Animal Studies, which is well-equipped for toxicological studies [37]. We will extend the resulting risk of bias analyses with two reporting questions, namely: “Was it mentioned that the study was blinded at any level?” and “Was it mentioned that the study was randomized at any level?”.

### Unit of analysis issues

If a consistent group of animals is studied (e.g., one cage), this will be used as the experimental unit. In case of single-housed mother-infant or mother-fetus studies, the mother will be considered to be the experimental unit. All dose levels will be converted to mg/m^3^ to allow comparison [38].

### Dealing with missing data

In case of missing data, this will be reported.

### Data synthesis and dose-response modeling

For the data synthesis, endpoints will be selected if they are considered adverse to the health of the animals and with relevance to human health. The studies will be ranked from the lowest to higher doses with regard to an adverse health effect for which the authors report a significant adverse effect compared to a reference dose (not necessarily a zero dose). If one good-quality/sensitive study is available for the most relevant low range of exposure that provides at least three dose groups (in addition to the control group) for the same outcome measure, we will fit a dose–response relationship to derive a point of departure (PoD) using the BMD application [39] and the most recent version of Benchmark Dose Software (https://www.epa.gov/bmds/about-benchmark-dose-software-bmds-version-3201). Subsequently, we will involve a panel of independent experts (see below) for a consensus decision on the choice of the endpoint considered as “critical,” taking into account the quality of the available studies. If multiple similar good-quality studies are available, we will consider the possibility of combining datasets from comparable studies that report on the same endpoint in a relevant exposure range. A panel of experts will be consulted to take a consensus decision on the benchmark response (BMR) and on the outcome measure chosen as the critical effect, including the critical effect size to discriminate a responder from a non-responder. We will assess how the choice of the mathematical model for curve fitting affects the estimated OEL, e.g., using the Akaike’s information criterion [40]. The benchmark dose lower bound (BMDL) from the best curve fit will be used to derive the health-based recommended OEL. If there are multiple datasets and/or multiple endpoints that relate to the same mode of action, the expert panel may consider to take the average BMDL to derive the health-based recommended OEL as this will provide more confidence in the recommendation [38].

### Panel of experts

A panel of experts will be invited to discuss and reach a consensus on critical choices to be made regarding the systematic review and OEL calculations. The experts have received the current systematic review protocol and discussed this during a meeting on December 7, 2021 (see meeting report in Supplementary material 5). The experts will also discuss the quality of the selected studies and discuss the choice of an adverse health outcome to be selected for the recommended health-based OEL. The committee will also discuss how to apply assessment factors (AF) to cover uncertainties in the extrapolation of animal data to humans. All invited experts will be asked to declare their interests.

## Discussion

This project aims to assess all available evidence on the effects of isoflurane in studies of controlled exposures in laboratory animals to derive a health-based recommended OEL for the workplace. For the study selection, we will consider studies in the low exposure range that is considered the most relevant to the occupational setting. This is operationalized as studies reporting on maintenance (rather than induction) doses below 1% [38]. If a study contains data points below this limit and also a few data points above this limit, the complete dataset will be included since we will rely on dose–response curve fitting as an important part of the data synthesis step (see below). Regarding the choice of endpoints, we will involve external experts to help us to take consensus decisions on which outcomes are considered adverse and what effect sizes are considered critical. It should be clear that effects observed in fetuses and newborns following exposure of the mother during pregnancy are not a direct result of maternal toxicity. This can only be considered if study authors monitor and report on the state of health of the dam. Hypoxia, hypercapnia, metabolic acidosis, metabolic alkalosis, ion imbalance, poor nutition, and physical stress have been implicated as contributiong to maternal effects that may result in anomalies in developmental toxicity study endpoints [41, 42]. The data synthesis will consist of dose–response modelling and leads to a derived dose that will be used as PoD to derive a health-based recommended OEL in humans. Uncertainties in the extrapolation from animals to humans will be addressed with AFs. These factors will be determined in accordance with current practices in chemical risk assessment [43–45]. For extrapolation of the available animal studies to humans, uncertainty factors will be applied for both intraspecies and interspecies uncertainties. The intraspecies aspect relates to the expected interindividual differences in sensitivity to isoflurane exposure within the population. For the general population, a variability over one order of magnitude is assumed, hence a factor 10 is usually adopted as AF to account for this uncertainty [43]. As workers are a subgroup within the general population restricted by age (excluding both young and elderly individuals), we consider it defendable to reduce this AF to a factor of 5 [43, 46]. Additional AFs may be considered, e.g., to extrapolate the exposure pattern available in selected studies to the reference time interval of an 8-h working day or 15 min for a short-term exposure window. The authors will involve a panel of experts to reach consensus decisions regarding justification of additional AFs.

### Supplementary Information

**Additional file 1: Supplementary material 1.** Scoping review. **Supplementary material 2, 3 and 4.** Search strategy. **Supplementary material 5.** Report of expert meeting.

## Data Availability

Raw data will be made available as a Supplementary file upon publication of the systematic review in an open-access journal.

## Abbrevations

AF: Assessment factor
BMD: Benchmark dose
BMDL: Benchmark dose lower bound
BMR: Benchmark response
CA: Chromosome aberration
CI: Confidence interval
GST: Glutathione-S-transferase
MAC: Minimum alveolar concentration
MN: Micronuclei
OEL: Occupational exposure level
OHAT: Office of Health Assessment and Translation
OR: Odds ratio
PoD: Point of departure
RR: Relative risk
SCE: Sister chromatide exchange
