# Supplementary material for: Establishing a health-based recommended occupational exposure limit for isoflurane using experimental animal data: a systematic review protocol
Source: Syst Rev. 2023 Sep 14;12:166. doi: 10.1186/s13643-023-02331-0 (PMC10503167; doi:10.1186/s13643-023-02331-0)
Supplement: Supplementary file 1 — Additional file 1: Supplementary material 1. Scoping review. Supplementary material 2, 3 and 4. Search strategy. Supplementary material 5. Report of expert meeting. [file 13643_2023_2331_MOESM1_ESM.docx]

**Supplementary material**

**Establishing a health-based recommended occupational exposure limit for isoflurane using experimental animal data – a systematic review protocol**

Fréderique Struijs, Carlijn R. Hooijmans, Marije Buijs, Albert Dahan, Sebastian Hoffmann, Romy Kiffen, Daniele Mandrioli, Julia Menon, Nel Roeleveld, Merel Ritskes-Hoitinga, Anne de Ruijter, Gert Jan Scheffer, Vivi Schlünssen, Paul T.J. Scheepers

**Corresponding author:**

Paul T.J. Scheepers, Radboud Institute for Biological and Environmental Sciences, Radboud University, Nijmegen, The Netherlands, tel +-31 - 653 748 401 e-mail [paul.scheepers@radboudumc.nl](about:blank)

Contents

[Supplementary material 1 - Scoping review 2](#_Toc144265136)

[Supplementary material 2 – Pubmed Search 24](#_Toc144265137)

[Supplementary material 3 – EMBASE Search 34](#_Toc144265138)

[Supplementary material 4 – Web of Science Search 41](#_Toc144265139)

[Supplementary material 5 - Report Expert Meeting 48](#_Toc144265140)

# Supplementary material 1 - Scoping review

**Scoping review of human and animal studies reporting on adverse outcomes for isoflurane**

Paul T.J. Scheepers, Julia M.L. Menon, Janne Swinkels, Judith van Luijk, Merel Ritskes-Hoitinga, Dept Health Evidence, Radboud Institute for Health Sciences, Radboudumc, Nijmegen

**Background**

This report describes a scoping review focusing on potential side effects of isoflurane that may be relevant for workers in healthcare facilities. Because of simultaneous use of multiple inhalation anesthetics often combined with intravenous medication, it is not feasible to evaluate the causality of isoflurane exposure with potential adverse health risk in real life exposure situations. Therefore, this scoping effort will focus on studies of side effects in experimentally well-controlled settings. Due to the limited availability and suitability of human data we will asp search for experimental animal studies. Observed adverse effects will then be evaluated with respect to their relevance for human health in an occupational setting.

**Objective**

Evaluation of the feasibility to derive an OEL for isoflurane based on published reports of toxicity testing of isoflurane in human and animal studies.

**Methods**

Before performing the search for animal studies, a dedicated search was performed to identify studies in workers exposed to isoflurane in an occupational setting. We also included human volunteer studies in a controlled experimental (laboratory environment). The aim of this study was to verify to what extent human studies support a causal relationship between exposure and health effects that are considered adverse. The search strategy for this dedicated search is presented in Appendix 1.

The search for animal data was designed and performed to retrieve all articles addressing isoflurane exposure in animals. The search was composed of two search components gathering several indexed terms for isoflurane and the Syrcle’s animal filter (De Vries et al., 2014) and were combined with the Boolean factor ‘AND’ (see Appendix 1). The search was executed the 15^th^ October 2019 in PubMed and generated 7810 articles. Non original studies identified, e.g. reviews, comments, letters, and editorials, were kept in a separate list (n=285) in Endnote. These 285 were identified as best as possible by searching their titles and notes. The articles that reported primary research data were subsequently exported to Excel and filtered in alphabetical order based by name of first author. Articles were assigned a random number via generation of a number list using an online random number generator (<https://www.random.org/sequences/>). These numbers were linked to the alphabetical list of references. Subsequently, the articles were ranked and the first 200 were selected for title abstract assessment in Rayyan.

The following criteria were used for title/abstract and full text screening:

*Title/Abstract phase*

1. In vivo experimental animal study
2. Study using isoflurane as intervention
3. Single exposure to isoflurane, so no other inhalation anaesthetics or use of intravenous anaesthetics or any other medication as pre-treatment or simultaneous exposure. Also, if only one group received if a single administration of isoflurane the study was included.
4. Administered as an inhalation anaesthetic. If the methods of administration were not specified, we assumed that it was by inhalation.

*Full text phase*

Same as above

1. Full text available
2. If foreign article, language is known or translation is available

After the full text screening the following data was extracted from the included articles:

- Author + year of publication
- Exposed population (species, sex, age)
- Concentration of isoflurane
- Unit of isoflurane
- Exposure pattern
- Outcome(s) assessed

When age was unclear, we approximated it by body weight of the animal if a growth curve was available in the provider’s website.

**Results**

The results from the search for relevant human studies will be discussed before turning to the retrieved studies following a search for experimental animal studies.

**Studies in humans**

The search for human data was performed on January 10, 2020 and resulted in 129 studies. Eleven studies were excluded because they were not relevant: one reported on animals and a second study used *in vitro* human cell systems. Of the human *in vivo* studies, five studies reported on the use of isoflurane in patients without addressing workers’ exposure. Four other studies related to occupational exposure but did not contain original data (were e.g. commentaries or reviews). Most of the remaining 118 studies reported on exposure to isoflurane used as inhalation anaesthetic in a healthcare facility. Only 25 studies reported additionally on endpoints relevant to human health. In **Table 1** the characteristics of these studies are presented. Below a brief description of these studies is provided and a synthesis of the evidence is presented

Newton and co-workers (1990) studied isoflurane in an experimental setting, involving healthy volunteers in a controlled inhalation exposure to evaluate effects on memory at high concentrations (0.1-0.4 x the Minimum Alveolar Concentration, MAC). All other studies reported on exposures in a healthcare facility. Most studies performed in a healthcare setting described the use of isoflurane combined with nitrous oxide and different other halogenated anaesthetics including halothane. Most studies did not provide quantitative data on exposure. Some studies described a method for exposure assessment but did not report any data in the abstract. Seven studies provided quantified isoflurane exposure levels based on workplace measurements using both direct reading methods and indirect methods (active air sampling using adsorbent tubes). Studies not showing any quantified data sometimes reported exposure as ‘low’ or ‘below recommended levels’. In 2018 Braz and co-workers studied exposure above ‘international recommendations’. One study presented levels of different inhalation anaesthetics in urine, including for isoflurane (Luccinni et al., 1997). Cope and co-workers presented exhaled breath concentrations as measure of internal exposure (Cope et al., 2002).

A wide range of endpoints was reported, all with toxicity parameters representing mechanisms considered to be relevant in humans. Most reported biomarkers were related to molecular events and cellular responses reflecting ‘early signals’ of subclinical and/or reversible changes including indicators of DNA damage. These studies were published over the past 20 years and typically performed in relatively small populations of less than 10 to several hundreds of study participants in the exposed group and a similar number as internal control group, usually healthcare workers with no known exposure. In addition, one large register-based epidemiological study was retrieved that looked at congenital malformations in a large cohort of 15,317 singleton live-borne children from 9,433 mothers working as registered nurses in Canada in 1990-2000 (Teschke et al., 2011). Exposure status was assessed by registered information on employer, type of healthcare facility, department, and position, combined with telephone interviews with knowledgeable healthcare personnel. Exposure to anaesthetic gases occurred in operation rooms and post-anaesthetic recovery rooms, while nitrous oxide was additionally used for pain relief in maternity wards. The use of several halogenated anaesthetic gasses increased over the study period, but isoflurane use was reported by 59-61% of the hospitals and nitrous oxide by 88-90% for 1990-2000. The researchers did not present data on miscarriages that have been linked to inhalation anaesthetics exposures in previous studies (see van Luijk et al. 2019). Congenital anomalies were associated with ‘ever’ maternal exposure to halogenated gases (OR=1.49, 95% CI: 1.04-2.13) and ‘probable’ maternal exposure to halogenated gases (OR=2.61, 95% CI: 1.31-5.18) and with ‘ever’ exposure to nitrous oxide (OR: 1.42, 95% CI: 1.05-1.94) and ‘probable’ exposure to nitrous oxide (OR=1.82, 95% CI: 1.11-2.99). The specific ORs for 'ever' and 'probable' maternal exposure to isoflurane were 1.63 (95% CI: 1.09-2.44) and 2.82 (1.37-5.82), respectively. The anomalies most frequently associated with halogenated anaesthetic gas exposures were those of the heart (OR=2.31, 95% CI: 1.07-4.97) and integument (OR=1.56, 95% CI: 1.53-8.32), with the ORs for isoflurane being 2.65 (95% CI: 1.16-6.02) and 3.91 (95% CI: 1.55-9.82). Isoflurane also seemed to be associated with congenital anomalies of the eyes (OR=2.78, 95% CI: 1.02-7.59) and ear, face, and neck (OR=3.05, 95% CI: 0.94-9.88). The strengths of this study were its large size and the cohort design using administrative records, which precludes selection and ascertainment bias as well as recall bias. Limitations were the fact that nurses were exposed to mixtures of inhalation anaesthetics, the small numbers of specific congenital anomalies which led to grouping of anomalies with potentially different aetiology, the lack of individual exposure measurements, the inability to pinpoint the presumed exposure to the sensitive time-window for the occurrence of congenital anomalies, and the lack of information on other occupational exposures and potential confounders. The latter may not have been very influential on the results as all comparisons were made within the nursing profession. As the exposure misclassification was most likely non-differential, it may have led to underestimation of the risk estimates and distortions in the shape of the dose-response relationships. Nevertheless, several clearly increased ORs were observed in this study, while the strengths of the associations increased with increasing likelihood of exposure.

For the ‘biomarker’ studies professionals in healthcare or animal facilities with exposure to inhalation anaesthetics were compared to professionals with similar job titles presumably non-exposed. Proietti et al., 2003 compared hospital staff working with open system to staff working with closed systems (Table 1). Only in one study medical residents were followed in time over periods of 8, 16 and 22 months of exposure and compared to control persons of similar age with no exposure (Costa Paes et al., 2014a). The largest group of thirteen studies aimed at biomarkers for genotoxicity in peripheral blood lymphocytes or exfoliated lymphocytes from buccal smears and reported on different indicators of DNA damage such as micronuclei (MN), sister chromatid exchanges (SCA), chromosome aberrations (CA) and comets. One study reported on different glutathione-S-transferase genotypes related to frequencies of MN and SCE (Kargar Shouroki et al., 2019). Two studies reported on a wide range of oxidative stress parameters in addition to DNA damage (Wronka-Nofer et al., 2012; Costa Paes et al., 2014a). In addition, some of these studies reported on cellular endpoints with relevance to apoptosis such as karyorrhexis and pyknosis. One study looked at neutrophil apoptosis (Goto et al., 2000). Some studies performed blood cell counts (e.g. Baregellini et al., 2001) and one study reported on pro-inflammatory biomarkers/cytokines (Chaoul et al., 2015). For most biomarkers, changes were observed for exposures in a range of 0.5 to 5 ppm for isoflurane often in the presence of much higher concentrations of nitrous oxide (12-580 ppm). Most of these changes were reported to be statistically significant different from biomarker levels observed in the group of controls, even for small numbers of 10-15 workers per group. Six studies reported on neuropsychological/neurobehavioral endpoints but no recent studies were retrieved after the study by Proieti et al. in 2003. One study reported on general haematological parameters (Amiri et al., 2018). Most of these studies observed inconsistent or no effects. Only Proietto et al., 2003 reported an effect on pshychomotor vigilance following an exposure of isoflurane of 11.1 ppm of personnel working with open systems compared 0.4 pm for closed systems. Nitrous oxide exposures were reported to be 301 ppm and 4.8 ppm, respectively.

In summary, only one epidemiology study was conducted in nurses that reported an increased risk of congenital anomalies at birth related to probability of exposures to halogenated inhalation anaesthetics including isoflurane. Attribution of anomalies specifically to isoflurane and sevoflurane was presented but difficult to verify based on information provided. Studies addressing neurobehavioral, neuropsychological endpoints did not observe consistent and strong indications of neurotoxicity in populations and exposures studied. In the past 15 years no new studies have addressed these neurology endpoints. Most studies and also most recent studies reported on changes in different indicators of DNA damage linked to combined exposure to either isoflurane with nitrous oxide or isoflurane combined with one or more other halogenated anaesthetics, including substances that have earlier been linked to DNA damage such as halothane. Few studies linked exposures, including isoflurane, to biomarkers of oxidative stress to pro-inflammatory markers and to genetic polymorphisms in GST iso-enzymes linked to DNA-damage. Most studies were conducted in Europe and reported these changes in biomarker levels at exposure levels below the recommended standards except for one study that was performed in Brazil.

Overall, the available studies provide a consistent pattern of changes in different indicators of DNA damage at relatively low exposures to inhalation anaesthetics of different professions in a healthcare compared to non-exposed controls with no known exposure in similar jobs. We did not find studies linking these exposures to adverse health effects except for one study suggesting a link with congenital anomalies in live-borne children of exposed nurses. Based on the available human data the causality of a relationship between exposure to sevoflurane and adverse health outcomes cannot be established with sufficient uncertainty. Other work-related exposures including nitrous oxide and other halogenated anaesthetics cannot be ruled out as contributing to these observed risks.

**Studies in animals**

An overview of the selection process is presented in Figure 1, using the Preferred Reporting Items for Systematic Reviews and Meta-Analyses (PRISMA) guidance. Our search generated 7,810 articles which were uploaded in Endnote. By searching in the titles and notes, we retrieved as best as possible non original studies e.g. reviews, letters. In total, 139 reviews and 126 other non-original studies were identified and removed from the selection of articles. From the 200 articles sample, only 58 were eligible for full text screening. All full text was found online, except for three that could not be found. Finally, 31 articles remained and split into 32 experiments. We describe in the section below the characteristics of these experiments in further detail.

**
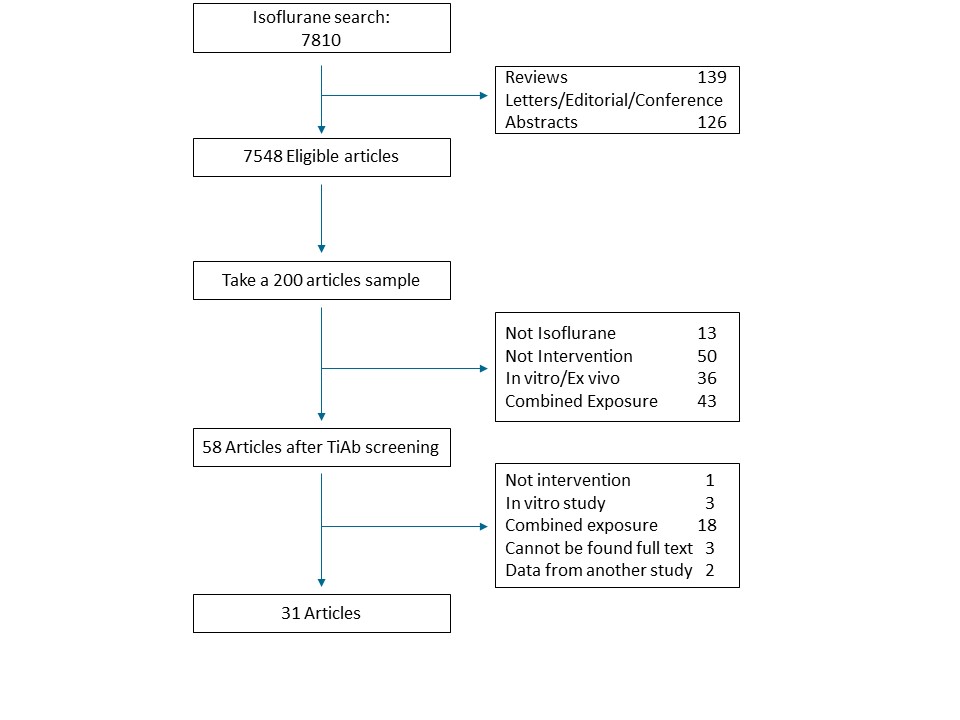
**

***Figure 1****: PRISMA diagram showing the selection of studies.*


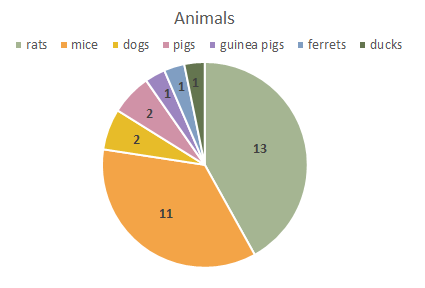


***Figure 2****. Overview of the animal species used Animal species*

Test animals were quite varied in the isoflurane exposure experiments (**Figure 2**). Rodents represented the majority of test animals (n=25), followed by mammals (n=3).


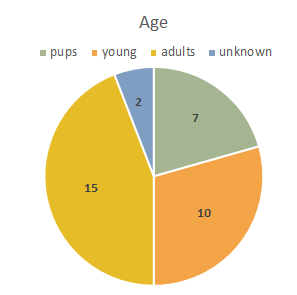


***Figure 3.*** *Overview of the age in the exposed animals*

The age of the animals was rather well reported, with a majority of adult animals (n=15) and young (n=10) (**Figure 3**). In five studies, we approximated the age of the animals by verifying their growth curve (n=5) on the website of the supplier. Additionally, in two studies, the age of the animals was a comparative variable. One study used pups, youth and old animals. Another study used both adult and juvenile animals.

*Sex of the exposed population*

Sex of the animals were rather well reported in our sample (**Figure 4**). Male animals represented the majority of the sample (n=14), followed by studies using both male and female animals (n=11). Studies using female animals only were very limited.


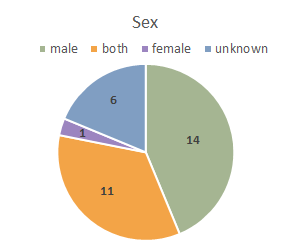


***Figure 4.*** *Overview of sex in exposed population*

*Exposure*

Isoflurane exposure ranged from 0.5-3.6% and 0.5-1.6 of the Maximum Alveolar Concentration, from which most studies used % as a measurement unit (n=27).

A majority of studies used only one isoflurane dose compared (by endpoints of interest) to controls. However, seven studies used several isoflurane doses, two (n=3), three (n=2), or four (n=2). In addition, for some papers, concentration was increased and/or decreased during the experiment. When a different dose was used for induction and for maintenance, we counted only the maintenance dose as the experimental dose.

We made a distinction between short (<1 hour), medium (1-2 hours), and long (>2 hours) exposure (**Figure 5**). If the exposure was precisely 2 hours, the entry ‘medium’ was used and if the exposure duration was ‘short’ but repeated on multiple days (e.g. 18 min each day for 7 or 14 days) it was placed under ‘long’ exposure duration.


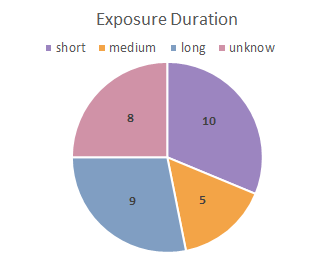


***Figure 5.*** *Overview of the exposure duration*

*Outcomes overview*

Outcomes were categorised in an overview of the most prevalent outcomes in the sample of included studies (**Table 2**). At this stage all reported outcomes are listed without a distinction in physiological parameter, biomarker or health outcome. In the full systematic review a similar list of outcomes will be presented to an expert panel to determine which outcomes are interpreted as ‘adverse’ and ‘health-related’ outcomes relevant to humans. In total, four categories were identified, namely neurological and neurodegenerative findings, hemodynamic variables, internal organs function and damage and physiology. Neurological and neurodegenerative findings were the most prevalent outcomes, with a focus on cognition and neurodegeneration.

**Conclusion**

The 31 studies eligible study identified in a random sample of 200 retrieved references, indicating that a predicted number of approximately 1210 studies (7810/200 x 31) will contain useful information to be able to determine the critical effect that can be used to derive an OEL. Considering the number of studies using at least two doses in our sample, it is expected that approximately 273 studies (7810/200 x 7) have tested isoflurane at two or more exposure levels that would support a dose-response assessment. In addition, it must be noted that for the scoping review only the PubMed database was explored, and that considerable number of relevant articles could be found from other databases. The results of this scoping review demonstrate that sufficient data is available to complete a full systematic review to derive an OEL.

**Acknowledgment**

For evaluation and interpretation of the study by Teschke *et al.* we have consulted dr. Nel Roeleveld of our department.

**References**

de Vries RB, Hooijmans CR, Tillema A, Leenaars M, Ritskes-Hoitinga M. Updated version of the Embase search filter for animal studies. Lab Anim. 2014;48(1):88. doi:10.1177/0023677213494374

van Luijk JAKR, Popa M, Swinkels J, et al. Establishing a health-based recommended occupational exposure limit for nitrous oxide using experimental animal data - A systematic review protocol. Environ Res. 2019;178:108711. doi:10.1016/j.envres.2019.108711

***Table 1:*** Overview of studies reporting on endpoints in workers and healthy volunteers (based on data from title and abstract).

| **Study author** | **Population (comparator)** | **Number index/controls** | **Inhalation anaesthetics (exposure level in ppm)** | **Type of endpoint** | **Endpoints** | **Outcome (statistically significant unless indicated otherwise)** |
| --- | --- | --- | --- | --- | --- | --- |
| Newton et al., 1990 | Healthy volunteers | 8 / - | Isoflurane (0.1, 0.2 and 0.4 MAC) | Neuropsycho-logical | Memory in a language text (not specified) | Both recall and recognition of neutral words was lost at 0.2 MAC and greater; the effect of attention was demonstrated by the memory of a ‘shock’ word by 4 out of 8 subjects at 0.2 MAC |
| Marraccini et al., 1992 | Workers with exposure to anaesthetic gases | 51 / - | Isoflurane (-)  Halothane (-)  nitrous oxide (-) | Neurotoxicity/ neurobehavioral | neurotoxicity or neurobehavioral | No important neurotoxicity or neurobehavioral problem observed at low concentration of anaesthetics |
| Sardas et al., 1992 | Anaesthetists, anaesthesia nurses and anaesthesia unit technicians (health unexposed controls) | 67 / 50 | Isoflurane (-), sevoflurane (-)  nitrous oxide (-) | Genotoxicity | Sister chromatid exchanges (SCE) in blood lymphocytes | Significant increase of SCEs in non-smokers compared to non-smoking controls |
| Luccini et al., 1997 | Operating theatre personnel (non-exposed hospital workers) | 112 /135 | Nitrous oxide in urine: 7.1 µg/l (P95 12.4, range 1.5-43) on the first and 7.8 µg/L (P95 21.5, range 1.0-73.3) on the last day of the week. Isoflurane in urine: 0.7 µg/L (P95 2.6, range 0-4.7) on the first day and 0.8 µg/L (P95 2.0, range 0-5.6) on the last day of the week | Neuropsycho-logical | Stroop colour Word reaction time test, EURO-QUEST neuropsychological symptoms questionnaire, WAIS block design sub test | No differences were observed |
| Sardas et al., 1998 | Anaesthetists, anaesthesia nurses and anaesthesia unit technicians (health unexposed controls) | 66 / not specified | Isoflurane (-), sevoflurane (-)  nitrous oxide (-) | Genotoxicity | Comets in blood lymphocytes using alkaline comet assay | Significant increase of Comets in non-smokers compared to non-smoking controls; A significant increase in the number of lymphocytes with DNA migration  was observed in operating room personnel |
| Hoerauf et al., 1999a | Non-smoking operating room personnel (non-smoking controls) | 10 /10 | Isoflurane (5.3)  Nitrous oxide (12.8) | Genotoxicity | Sister chromatid exchanges (SCE) and micronuclei (MN) in blood lymphocytes | Mean (sd) of SCE higher (10.2  (1.9) vs. 7.4 (2.4) (p = 0.036) Mean (sd) of MN was higher (8.7 (2.9) vs. 6.8 (2.5)) but not significant (p = 0.10). |
| Hoerauf et al., 1999b | Non-smoking operating room personnel (non-smoking controls) | 27 / 27 | Isoflurane (0.5)  Nitrous oxide (11.8) | Genotoxicity | Sister chromatid exchanges (SCE) and micronuclei (MN) in blood lymphocytes | SCEs were significantly increased: mean±sd = 9.0±1.3 compared to 8.0±1.4 in controls (P < 0.05). Anaesthetic gases may cause  genetic damage comparable with smoking 11-20 cigarettes per day. |
| Goto et al, 2000 | Healthcare workers (unexposed volunteers) | 20 / 10 | Isoflurane, sevoflurane  nitrous oxide described as ‘well below recommended limits’ | Cytotoxicity | Neutrophil apoptosis | Inhibition of neutrophil apoptosis at 24 h culture compared to controls |
| Proietti et al., 2000 | Operating room personnel | 300 / - | Isoflurane (-)  Nitrous oxide (-) | Neurobehavioral | Unspecified blood-based biomarkers and an unspecified method of determining anxiety | Blood tests did not have a valid index of possible damage. A ‘large percentage’ of anaesthetists were found to have anxiety |
| Baregellini et al., 2001 | Anaesthetists (non-exposed physicians matched by gender age and job title) | 51 / 51 | Isoflurane (-)  Nitrous oxide (-) | Immunology | Lymphocyte T helper (TH) cell counts, Lymphocyte subpopulations and natural killer (NK) cytotoxicity | Decrease of T-cells (CD3). Numbers of NK cells (CD16+ and CD3-) increased. The TH cell count was reduced with increased individual exposure score based on number of working days and also with levels of exposures. |
| Bozkurt et al., 2002 | No-smoking anaesthetists (matched with non-smoking physicians without exposure) | 16 / 16 | Not specified (‘the nature of our anaesthesia practice suggests exposure was likely to be low’). | Genotoxicity | Sister chromatid exchanges (SCE) and high frequency SCEs (HFCs) in peripheral lymphocytes | 6.6±2.4% SCEs and 12.2±15.9% HFCs were not statistically significantly greater than in the reference group (5.2±1.6 and 5.9±10.0). |
| Cope et al., 2002 | Nurses working in the post-anaesthesia care unit (post-shift compared to pre-shift within the same person) | No specified | Isoflurane measured in exhaled breath (43±30 ppb in pre-shift sample and 124±57 ppb in post-shift same) | Neurotoxicity | Central neuro control of breathing | Central neuro-respiratory activity was depressed from pre-shift to post-shift on Monday but not on Friday. |
| Proietti et al., 2003 | Anaesthesiologists (comparison between open and closed system) | Not specified | Isoflurane (11.1 in open and 0.4 closed system)  Nitrous oxide (301 in open and 4.83 in closed system) | Neurobehavioral | Psychomotor vigilance | The mean of the reaction time was significantly higher (p < 0.01) during work with the open system compared to work in low flow at the end of the first weekday shift and at the end of the last weekday shift. |
| Wronska-Nofer et al., 2009 | Female nurses and male anaesthesiologists (unexposed female nurses and male doctors) | 84 / 83 | Isoflurane (-), sevoflurane (-),  nitrous oxide (-) (exposure measured but not reported in abstract) | Genotoxicity | Comets | Positive correlation between the DNA damage and exposure to nitrous oxide. No correlation between genotoxic effects and concentrations of sevoflurane and isoflurane. Genetic injury was especially aggravated among nurses and anaesthesiologists exposed to nitrous oxide >180 mg/m^3^ |
| Teschke et al., 2011 | Registered nurses | 15,317 live-borne children of 9,433 mothers, 1,079 had congenital anomalies | Nitrous oxide (-)  Halogenated gases including:  Isoflurane (-) sevoflurane (-)  Halothane (-) | Reproductive and developmental | Congenital anomalies | Anomalies were associated with ever and probable maternal exposure to halogenated gases (ORs: 1.49, 95% CI: 1.04-2.13; and 2.61, 95% CI: 1.31-5.18, resp.) and to nitrous oxide (ORs: 1.42, 95% CI: 1.05-1.94; and 1.82, 95%  CI: 1.11-2.99). Anomalies most frequently associated with exposure were those of  the heart (OR, halogenated gases: 2.31, 95% CI: 1.07-4.97) and integument (OR, halogenated gases: 3.56, 95% CI: 1.53-8.32; OR, nitrous oxide: 3.02, 95% CI:  1.37-6.64). Gases most frequently associated with anomalies were halothane (predominantly used early in the study period), isoflurane, and sevoflurane (predominantly used later in the period). |
| Wronska-Nofer et al., 2012 | Nurses (unexposed nurses) | 36 / 36 | Isoflurane, sevoflurane, nitrous oxide | Oxidative stress and DNA-damage | Thiobarbituric acid reactive substances (TBARS). α-tocopherol, glutathione peroxidase (GPX) and 8-isoprostaglandin F(2α) (8-iso-PGF(2α)) in urine | Positive correlation between  the oxidative DNA damage and nitrous oxide. No association between genotoxic effects and sevoflurane or isoflurane. ROS generation and plasma and urine concentrations of TBARS and 8-iso-PGF(2α) were elevated compared to controls whereas GPX activity was reduced compared to controls. |
| Costa Paes et al., 2014a | Anaesthesiology and surgery medical residents were followed up 8, 16 and 22 months of exposure (young adult with no exposure) | 15 /15 | Isoflurane (and to a lesser extent also to sevoflurane and nitrous oxide) | Oxidative stress and genotoxicity | Glutathione peroxidase (GPX) and, superoxide dismutase (SOC) and catalase (CAT) Comets | DNA damage increased at 8, 16 and 22 M evaluated in the exposed group; plasma thiols increased at 22 months of exposure and GPX was higher at 16 and 22 months of exposure. |
| Chaoul et al., 2015 | Operating room personnel (medical personnel with no known exposure) | 15 / 15 | Mixture of anaesthetic gases (not specified) | Immunology | Pro-inflammatory cytokines | Increase in IL-8 in high exposed personnel with exposure for a short period |
| Souza et al., 2016 | Anaesthesiologists (matched non-exposed controls) | Not specified | Isoflurane (-), sevoflurane (-),  desflurane (-)  nitrous oxide (>170) | Genotoxicity/genome instability/  Cytotoxicity/ proliferative changes | Micronuclei (MN), karyorrhexis, pyknosis in lymphocytes from exfoliated exhaled buccal samples | Lower basal cell count; no differences in lymphocyte DNA damage but higher frequency of MN, karyorrhexis and pyknosis compared to controls |
| Szyfter et al., 2016 | Nurses and anaesthesiologists | 100 | Isoflurane, halothane and ‘others’ (n/a) | Genotoxicity | Comets in blood lymphocytes | No difference in comet frequency compared with ‘other groups’; only difference in ‘cumulative DNA lesions’ in anaesthesiologists |
| Amiri et al., 2018 | Operating room personnel (unexposed nurses) | 52 / 52 | Isoflurane (2.40)  sevoflurane (0.18)  nitrous oxide (850.92) | Haematology | Red blood cell count  Haemoglobin (Hb)  Mean corpuscular Hb  Haematocrit | Lower red blood cell count (Hb)  mean corpuscular Hb, mean corpuscular Hb concentration and haematocrit compered to controls |
| Aun et al. 2018 | Young physicians during first years of residence | 26 / - | Isoflurane (-), sevoflurane (-), desflurane (-),  nitrous oxide (-) | Genotoxicity and cytotoxicity | Comets and apoptosis; loss of mitochondrial membrane potential | Not induce either cytotoxicity or genotoxicity in mononuclear cells over the first year of residence |
| Braz et al., 2018 | Physicians (medical residents) | 60 / not specified | Isoflurane, sevoflurane, nitrous oxide (higher than ‘international recommendations’) | Genotoxicity | Micronuclei, karyorrhexis, pyknosis and differentiated cells in exfoliated buccal cells | Lower frequencies of basal cells,  Higher frequencies of micronuclei; pyknosis, Karyorrhexis frequencies were higher in anaesthesiologists |
| Kargar Shouroki et al., 2019 | Operating room personnel (unexposed nurses) | 60 / 60 | Isoflurane (2.40 ± 0.86)  Sevoflurane (0.18 ± .14)  Nitrous oxide (850.92 ± 919.78) | Genotoxicity and  Genotyping (genetic polymorphisms) | Micronuclei (MN); chromosome aberrations (CA); glutathione-S-transferases (GST): GSTM1 GSTP1, GSTT1 | Higher frequencies of MNs and CA compared to controls; Differences in MN depending on exposure, gender and genotype |

***Table 2:*** *Overview of outcomes reported in the study sample (n=31). For each outcome the number of available studies is given in parenthesis.*

| **Category** | **Sub-Category** | **Test/Pathways** |
| --- | --- | --- |
| Neurological and Neurodegenerative Findings (16) | Cognition (9) | Moris Maze Water (6) |
|  |  | Fear conditioning (3) |
|  |  | Open Field (2) |
|  |  | Circadian Rhythm measurement (2) |
|  |  | Elevated plus maze (1) |
|  |  | Y Maze (1) |
|  |  | Barne maze test (1) |
|  |  | Forced swim test (1) |
|  |  | Nest construction (1) |
|  |  | Saccharin preference test (1) |
|  |  | Light-dark box (1) |
|  |  | Object recognition test (1) |
|  | Brain function (5) | Brain cell histology and development (3), neuronal action potential (1) |
|  | Neurodegeneration (5) | Neuroapoptosis (7) |
|  |  | Neuroinflammation (3) |
|  | Brain Metabolism (2) | Levels of dopamine and its metabolite in the striatum (1), glutamate and GABA levels |
| Hemodynamic Variables (14) | Physiological variable (11) | Heart rate, mean arterial blood pressure, pH, metabolic rate of O2, Co2 |
|  | Test of MAC/efficacity of anaesthesia (4) | No specific information |
| Internal organs function and damage (4) | Lung histology and function (2) | No specific information |
|  | Lung inflammation (1) | No specific information |
|  | Liver function (2) | Level of CoA and acetyl CoA, triglycerides, and free fatty acids (1) |
|  | Liver damage (1) | Alanine aminotransferase metabolism (1), hydrophilic compounds (1) |
|  | Muscle damage (1) | Creatinine pathway (1) |
| Physiology (4) | Pituitary and adrenocortical hormones (1) | Stress pathway (1) |
|  | Body weight (2) | No specific information |
|  | Molecular levels (2) | plasma level of beta-hydroxybutyrate and acetoacetate (1), malondialdehyde levels (1) |

***Appendix 1:*** Detailed search strings

**Human studies**

((Isoflurane [MeSH] OR isoflurane[tiab] OR isofluran[tiab] OR No “26675 46 7” [tiab] OR “26675 46 7 “[RN] OR “1 chloro 2,2,2 trifluoroethyl difluoromethyl ether” [tiab] OR “2 chloro 2 difluoromethoxy 1,1,1 trifluoroethane”[tiab] OR forane[tiab] OR forene [tiab]) AND (Humans[MeSH] OR Health Personnel[MeSH] OR Dentists[MeSH] OR Nurse Midwives[MeSH] OR staff[tiab] OR personnel[tiab] OR employee[tiab] OR nurse[tiab] OR nurses[tiab] OR doctor[tiab] OR doctors[tiab] OR physician[tiab] OR physicians[tiab] OR anaesthesiologist[tiab] OR anaesthesiologists[tiab] OR anesthesiologist[tiab] OR anesthesiologists[tiab] OR anesthetist[tiab] OR anesthetists[tiab] OR anaesthetist[tiab] OR anaesthetists[tiab] OR pediatrician[tiab] OR pediatricians[tiab] OR paediatrician[tiab] OR paediatricians[tiab] OR assistant[tiab] OR assistants[tiab] OR midwife[tiab] OR midwives[tiab] OR dentist[tiab] OR dentists[tiab] OR women[tiab] OR men[tiab] OR volunteer[tiab] OR volunteers[tiab] OR Healthcare Provider[tiab] OR healthcare providers[tiab] OR healthcare worker[tiab] OR healthcare workers[tiab])) NOT (Occupational exposure [MeSH] OR “Threshold Limit Values” [MeSH] OR (Occupational [tiab] AND (exposure [tiab] OR exposures [tiab])) OR (Maximum [tiab] AND (Allowable [tiab] OR permissible [tiab]) AND (Concentration [tiab] OR concentrations [tiab])) OR OEL [tiab] OR OELs [tiab] OR MPEL [tiab] OR MPELs [tiab])

**Animal studies**

Isoflurane [MeSH] OR isoflurane[tiab] OR isofluran[tiab] OR No 26675 46 7 [tiab] OR 26675 46 7 [RN] OR “1 chloro 2,2,2 trifluoroethyl difluoromethyl ether” [tiab] OR “2 chloro 2 difluoromethoxy 1,1,1 trifluoroethane” [tiab] OR forane[tiab] OR forene [tiab]

AND Syrcle’s animal filter (de Vries et al., 2014)

# Supplementary material 2 – Pubmed Search

isoflurane [MESH] OR isofluran* [tiab] OR isofluorane [tiab] OR isoba [tiab] OR isofor [tiab] OR isoforine [tiab] OR isoflo [tiab] OR isorrane [tiab] OR isorane [tiab] OR sofloran [tiab] OR forthane [tiab] OR forane [tiab] OR forene [tiab] OR aerrane [tiab] OR terrel [tiab] OR 1 chloro 2,2,2 trifluoroethyl difluoromethyl ether [tiab] OR 26675-46-7 [tiab] OR 26675-46-7 [RN]

AND

(animal experimentation[MeSH] OR models, animal[MeSH] OR Animals[Mesh:noexp] OR animal population groups [MeSH] OR chordata[MeSH Terms:noexp] OR vertebrates[MeSH Terms:noexp] OR amphibians[MeSH] OR birds[MeSH] OR fishes[MeSH] OR reptiles[MeSH] OR mammals[MeSH Terms:noexp] OR primates[MeSH Terms:noexp] OR eutheria[MeSHTerms:noexp] OR artiodactyla[MeSH] OR carnivore[MeSH] OR cephalopoda[MeSH] OR cetacea[MeSH] ORchiroptera[MeSH] OR elephants[MeSH] OR hyraxes[MeSH] OR insectivora[MeSH] OR lagomorpha[MeSH] OR marsupialia[MeSH] OR monotremata[MeSH] OR perissodactyla[MeSH] OR Proboscidea Mammal[MeSH Terms:noexp] OR rodentia[MeSH] OR scandentia[MeSH] OR sirenia[MeSH] OR cingulata[MeSH] OR haplorhini[MeSH Terms:noexp] OR strepsirhini[MeSH] OR platyrrhini[MeSH] OR tarsii[MeSH] OR catarrhini[MeSH Terms:noexp] OR cercopithecidae[MeSH] OR hylobatidae[MeSH] OR hominidae[MeSH Terms:noexp] OR gorilla gorilla[MeSH] OR pan paniscus[MeSH] OR pantroglodytes[MeSH] OR pongo[MeSH]) OR ((rat[tiab] OR rats[tiab] OR animal[tiab] OR animals[tiab] OR mice[tiab] OR in vivo[tiab] OR mouse[tiab] OR rabbit[tiab] OR rabbits[tiab] OR murine[tiab] OR pig[tiab] OR pigs[tiab] OR dog[tiab] OR dogs[tiab] OR bovine[tiab] OR fish[tiab] OR vertebrate[tiab] OR vertebrates[tiab] OR cat[tiab] OR cats[tiab] OR rodent[tiab] OR rodents[tiab] OR mammal[tiab] OR mammals[tiab] OR chicken[tiab] OR chickens[tiab] OR monkey[tiab] OR monkeys[tiab] OR sheep[tiab] OR canine[tiab] OR canines[tiab] OR porcine[tiab] OR cattle[tiab] OR bird[tiab] OR birds[tiab] OR hamster[tiab] OR hamsters[tiab] OR primate[tiab] OR primates[tiab] OR cow[tiab] OR cows[tiab] OR chick[tiab] OR horse[tiab] OR horses[tiab] OR avian[tiab] OR avians[tiab] OR calf[tiab] OR swine[tiab] OR swines[tiab] OR xenopus[tiab] OR turkeys[tiab] OR bear[tiab] OR bears[tiab] OR frog[tiab] OR frogs[tiab] OR zebrafish[tiab] OR goat[tiab] OR goats[tiab] OR equine[tiab] OR calves[tiab] OR poultry[tiab] OR macaque[tiab] OR macaques[tiab] OR mole[tiab] OR moles[tiab] OR ovine[tiab] OR lamb[tiab] OR lambs[tiab] OR fishes[tiab] OR diptera[tiab] OR amphibian[tiab] OR amphibians[tiab] OR snake[tiab] OR snakes[tiab] OR ruminant[tiab] OR ruminants[tiab] OR hen[tiab] OR hens[tiab] OR piglet[tiab] OR piglets[tiab] OR feline[tiab] OR felines[tiab] OR simian[tiab] OR simians[tiab] OR laevis[tiab] OR trout[tiab] OR trouts[tiab] OR teleost[tiab] OR teleosts[tiab] OR salmon[tiab] OR salmons[tiab] OR seal[tiab] OR seals[tiab] OR bull[tiab] OR bulls[tiab] OR ewe[tiab] OR ewes[tiab] OR hedgehog[tiab] OR hedgehogs[tiab] OR macaca[tiab] OR macacas[tiab] OR proteus[tiab] OR pigeon[tiab] OR pigeons[tiab] OR bat[tiab] OR bats[tiab] OR duck[tiab] OR ducks[tiab] OR chimpanzee[tiab] OR chimpanzees[tiab] OR baboon[tiab] OR baboons[tiab] OR deer[tiab] OR rana[tiab] OR ranas[tiab] OR carp[tiab] OR carps[tiab] OR heifer[tiab] OR swallow[tiab] OR swallows[tiab] OR lizard[tiab] OR lizards[tiab] OR canis[tiab] OR sow[tiab] OR sows[tiab] OR cynomolgus[tiab] OR quail[tiab] OR quails[tiab] OR reptile[tiab] OR reptiles[tiab] OR turtle[tiab] OR turtles[tiab] OR buffalo[tiab] OR gerbil[tiab] OR gerbils[tiab] OR boar[tiab] OR boars[tiab] OR squirrel[tiab] OR squirrels[tiab] OR oncorhynchus[tiab] OR mus[tiab] OR toad[tiab] OR toads[tiab] OR fowl[tiab] OR fowls[tiab] OR rerio[tiab] OR danio[tiab] OR ara[tiab] OR aras[tiab] OR musculus[tiab] OR tadpole[tiab] OR tadpoles[tiab] OR mulatta[tiab] OR salmo[tiab] OR ram[tiab] OR eagle[tiab] OR eagles[tiab] OR ferret[tiab] OR ferrets[tiab] OR goldfish[tiab] OR catfish[tiab] OR whale[tiab] OR whales[tiab] OR fox[tiab] OR foxes[tiab] OR ape[tiab] OR apes[tiab] OR elephant[tiab] OR elephants[tiab] OR bos[tiab] OR marmoset[tiab] OR marmosets[tiab] OR cod[tiab] OR cods[tiab] OR shark[tiab] OR sharks[tiab] OR wolf[tiab] OR eel[tiab] OR eels[tiab] OR auratus[tiab] OR rattus[tiab] OR zebra[tiab] OR zebras[tiab] OR tilapia[tiab] OR tilapias[tiab] OR gilt[tiab] OR camel[tiab] OR camels[tiab] OR squid[tiab] OR gallus[tiab] OR marsupial[tiab] OR marsupials[tiab] OR vole[tiab] OR voles[tiab] OR fascicularis[tiab] OR ovis[tiab] OR salmonid[tiab] OR salmonids[tiab] OR tiger[tiab] OR tigers[tiab] OR dolphin[tiab] OR dolphins[tiab] OR robin[tiab] OR robins[tiab] OR carpio[tiab] OR opossum[tiab] OR opossums[tiab] OR cyprinus[tiab] OR salamander[tiab] OR salamanders[tiab] OR felis[tiab] OR mink[tiab] OR minks[tiab] OR swan[tiab] OR swans[tiab] OR norvegicus[tiab] OR bufo[tiab] OR torpedo[tiab] OR bass[tiab] OR lamprey[tiab] OR lampreys[tiab] OR sus[tiab] OR python[tiab] OR pythons[tiab] OR tetrapod[tiab] OR tetrapods[tiab] OR shrew[tiab] OR shrews[tiab] OR lion[tiab] OR lions[tiab] OR hog[tiab] OR hogs[tiab] OR songbird[tiab] OR songbirds[tiab] OR oreochromis[tiab] OR starling[tiab] OR starlings[tiab] OR caprine[tiab] OR carassius[tiab] OR owl[tiab] OR owls[tiab] OR newt[tiab] OR newts[tiab] OR papio[tiab] OR scrofa[tiab] OR hare[tiab] OR hares[tiab] OR gorilla[tiab] OR gorillas[tiab] OR flounder[tiab] OR flounders[tiab] OR goose[tiab] OR herring[tiab] OR herrings[tiab] OR therian[tiab] OR buffaloes[tiab] OR canary[tiab] OR sparrow[tiab] OR sparrows[tiab] OR microtus[tiab] OR octopus[tiab] OR troglodytes[tiab] OR tuna[tiab] OR amphibia[tiab] OR chinchilla[tiab] OR chinchillas[tiab] OR ide[tiab] OR oryzias[tiab] OR cervus[tiab] OR kangaroo[tiab] OR kangaroos[tiab] OR armadillo[tiab] OR armadillos[tiab] OR callithrix[tiab] OR pan troglodytes[tiab] OR saimiri[tiab] OR cichlid[tiab] OR cichlids[tiab] OR donkey[tiab] OR donkeys[tiab] OR bream[tiab] OR char[tiab] OR chars[tiab] OR finch[tiab] OR raccoon[tiab] OR raccoons[tiab] OR bothrops[tiab] OR anguilla[tiab] OR perch[tiab] OR cricetus[tiab] OR seabird[tiab] OR seabirds[tiab] OR buck[tiab] OR bucks[tiab] OR naja[tiab] OR coturnix[tiab] OR salmonids[tiab] OR geese[tiab] OR minnow[tiab] OR minnows[tiab] OR raptor[tiab] OR raptors[tiab] OR merione[tiab] OR meriones[tiab] OR rodentia[tiab] OR elaphus[tiab] OR amniote[tiab] OR amniotes[tiab] OR elasmobranch[tiab] OR emu[tiab] OR emus[tiab] OR peromyscus[tiab] OR hominid[tiab] OR hominids[tiab] OR bubalus[tiab] OR crotalus[tiab] OR gull[tiab] OR gulls[tiab] OR anas[tiab] OR anura[tiab] OR lemur[tiab] OR lemurs[tiab] OR crow[tiab] OR crows[tiab] OR camelus[tiab] OR gibbon[tiab] OR gibbons[tiab] OR waterfowl[tiab] OR parrot[tiab] OR parrots[tiab] OR eels[tiab] OR cob[tiab] OR stickleback[tiab] OR sticklebacks[tiab] OR columba[tiab] OR mesocricetus[tiab] OR ambystoma[tiab] OR raven[tiab] OR ravens[tiab] OR gadus[tiab] OR penguin[tiab] OR penguins[tiab] OR orangutan[tiab] OR orangutans[tiab] OR sturgeon[tiab] OR sturgeons[tiab] OR cuniculus[tiab] OR aves[tiab] OR virginianus[tiab] OR cephalopod[tiab] OR cephalopods[tiab] OR cebus[tiab] OR sparus[tiab] OR tortoise[tiab] OR tortoises[tiab] OR guttata[tiab] OR morhua[tiab] OR unguiculatus[tiab] OR dogfish[tiab] OR vulpes[tiab] OR mallard[tiab] OR mallards[tiab] OR apodemus[tiab] OR alligator[tiab] OR alligators[tiab] OR oryctolagus[tiab] OR llama[tiab] OR llamas[tiab] OR reindeer[tiab] OR mustela[tiab] OR duckling[tiab] OR ducklings[tiab] OR wolves[tiab] OR sander[tiab] OR amazona[tiab] OR zebu[tiab] OR badger[tiab] OR badgers[tiab] OR dove[tiab] OR doves[tiab] OR ictalurus[tiab] OR capra[tiab] OR capras[tiab] OR equus[tiab] OR camelid[tiab] OR camelids[tiab] OR poecilia[tiab] OR mule[tiab] OR mules[tiab] OR perciformes[tiab] OR salvelinus[tiab] OR labrax[tiab] OR cyprinidae[tiab] OR ariidae[tiab] OR crocodile[tiab] OR crocodiles[tiab] OR fundulus[tiab] OR dicentrarchus[tiab] OR clarias[tiab] OR cercopithecus[tiab] OR chiroptera[tiab] OR alpaca[tiab] OR alpacas[tiab] OR pike[tiab] OR pikes[tiab] OR paralichthys[tiab] OR puma[tiab] OR pumas[tiab] OR didelphis[tiab] OR pisces[tiab] OR macropus[tiab] OR triturus[tiab] OR bison[tiab] OR bisons[tiab] OR epinephelus[tiab] OR gasterosteus[tiab] OR panthera[tiab] OR acipenser[tiab] OR mackerel[tiab] OR mackerels[tiab] OR tamarin[tiab] OR tamarins[tiab] OR ostrich[tiab] OR anolis[tiab] OR vervet[tiab] OR vervets[tiab] OR wallaby[tiab] OR glareolus[tiab] OR beaver[tiab] OR beavers[tiab] OR dromedary[tiab] OR catus[tiab] OR killifish[tiab] OR pimephales[tiab] OR promelas[tiab] OR aotus[tiab] OR phoca[tiab] OR panda[tiab] OR pandas[tiab] OR porpoise[tiab] OR porpoises[tiab] OR myotis[tiab] OR yak[tiab] OR yaks[tiab] OR agkistrodon[tiab] OR vipera[tiab] OR otter[tiab] OR otters[tiab] OR turbot[tiab] OR turbots[tiab] OR squamate[tiab] OR carnivora[tiab] OR mullet[tiab] OR mullets[tiab] OR hawk[tiab] OR hawks[tiab] OR taeniopygia[tiab] OR seahorse[tiab] OR seahorses[tiab] OR poecilia reticulata[tiab] OR falcon[tiab] OR falcons[tiab] OR prosimian[tiab] OR prosimians[tiab] OR parus[tiab] OR perca[tiab] OR fingerling[tiab] OR fingerlings[tiab] OR antelope[tiab] OR antelopes[tiab] OR tupaia[tiab] OR passeriformes[tiab] OR sepia[tiab] OR saguinus[tiab] OR coyote[tiab] OR coyotes[tiab] OR pongo[tiab] OR meleagris[tiab] OR reptilia[tiab] OR lepus[tiab] OR psittacine[tiab] OR hagfish[tiab] OR warbler[tiab] OR warblers[tiab] OR russell's viper[tiab] OR russell's vipers[tiab] OR smolt[tiab] OR smolts[tiab] OR budgerigar[tiab] OR sardine[tiab] OR sardines[tiab] OR cavia[tiab] OR cavias[tiab] OR hyla[tiab] OR pleurodeles[tiab] OR siluriformes[tiab] OR great tit[tiab] OR great tits[tiab] OR guppy[tiab] OR bonobo[tiab] OR bonobos[tiab] OR rutilus[tiab] OR trichosurus[tiab] OR muridae[tiab] OR phodopus[tiab] OR channa[tiab] OR squalus[tiab] OR lynx[tiab] OR sturnus[tiab] OR petromyzon[tiab] OR vitulina[tiab] OR monodelphis[tiab] OR cuttlefish[tiab] OR adder[tiab] OR adders[tiab] OR lepomis[tiab] OR canaria[tiab] OR gambusia[tiab] OR guppies[tiab] OR xiphophorus[tiab] OR flatfish[tiab] OR koala[tiab] OR koalas[tiab] OR labeo[tiab] OR stingray[tiab] OR stingrays[tiab] OR chelonia[tiab] OR lampetra[tiab] OR spermophilus[tiab] OR crocodilian[tiab] OR passer domesticus[tiab] OR sciurus[tiab] OR artiodactyla[tiab] OR ranidae[tiab] OR corvus[tiab] OR necturus[tiab] OR platypus[tiab] OR canaries[tiab] OR bovid[tiab] OR lagopus[tiab] OR trimeresurus[tiab] OR gariepinus[tiab] OR marten[tiab] OR martens[tiab] OR drosophilidae[tiab] OR mugil[tiab] OR sunfish[tiab] OR porcellus[tiab] OR cypriniformes[tiab] OR alouatta[tiab] OR scophthalmus[tiab] OR anser[tiab] OR electrophorus[tiab] OR putorius[tiab] OR iguana[tiab] OR iguanas[tiab] OR lama[tiab] OR lamas[tiab] OR takifugu[tiab] OR circus[tiab] OR eptesicus[tiab] OR flycatcher[tiab] OR galago[tiab] OR galagos[tiab] OR trachemys[tiab] OR lungfish[tiab] OR characiformes[tiab] OR shorebird[tiab] OR shorebirds[tiab] OR giraffe[tiab] OR giraffes[tiab] OR micropterus[tiab] OR scyliorhinus[tiab] OR cichlidae[tiab] OR loligo[tiab] OR porcupine[tiab] OR porcupines[tiab] OR chub[tiab] OR chubs[tiab] OR solea[tiab] OR pleuronectes[tiab] OR hylidae[tiab] OR viperidae[tiab] OR echis[tiab] OR sorex[tiab] OR anchovy[tiab] OR lagomorph[tiab] OR ostriches[tiab] OR vulture[tiab] OR vultures[tiab] OR whitefish[tiab] OR araneus[tiab] OR jird[tiab] OR jirds[tiab] OR tern[tiab] OR esox[tiab] OR drake[tiab] OR drakes[tiab] OR elapidae[tiab] OR gallopavo[tiab] OR chordata[tiab] OR myodes[tiab] OR caretta[tiab] OR serinus[tiab] OR grouse[tiab] OR misgurnus[tiab] OR meles[tiab] OR blackbird[tiab] OR blackbirds[tiab] OR coregonus[tiab] OR bobwhite[tiab] OR bobwhites[tiab] OR heteropneustes[tiab] OR mammoth[tiab] OR mammoths[tiab] OR turdus[tiab] OR rhinella[tiab] OR ateles[tiab] OR characidae[tiab] OR clupea[tiab] OR bungarus [tiab] OR brill[tiab] OR struthio camelus[tiab] OR sloth[tiab] OR sloths[tiab] OR pteropus[tiab] OR sculpin[tiab] OR anthropoids[tiab] OR pollock[tiab] OR pollocks[tiab] OR morone[tiab] OR pan paniscus[tiab] OR litoria[tiab] OR chipmunk[tiab] OR chipmunks[tiab] OR balaenoptera[tiab] OR marmota[tiab] OR melopsittacus[tiab] OR hyrax[tiab] OR lemming[tiab] OR lemmings[tiab] OR halibut[tiab] OR hylobates[tiab] OR lates[tiab] OR caiman[tiab] OR caimans[tiab] OR sigmodon[tiab] OR stenella[tiab] OR barbel[tiab] OR barbels[tiab] OR sterna[tiab] OR parakeet[tiab] OR parakeets[tiab] OR phocoena[tiab] OR leptodactylus[tiab] OR canidae[tiab] OR buteo[tiab] OR harengus[tiab] OR gopher[tiab] OR gophers[tiab] OR marmot[tiab] OR marmots[tiab] OR gosling[tiab] OR goslings[tiab] OR platichthys[tiab] OR gar[tiab] OR gars[tiab] OR sebastes[tiab] OR marsupialia[tiab] OR notophthalmus[tiab] OR gazelle[tiab] OR gazelles[tiab] OR insectivora[tiab] OR paridae[tiab] OR felidae[tiab] OR russula[tiab] OR galliformes[tiab] OR bombina[tiab] OR colobus [tiab] OR echidna[tiab] OR echidnas[tiab] OR seabass[tiab] OR syncerus[tiab] OR plaice[tiab] OR blue tit[tiab] OR blue tits[tiab] OR pagrus[tiab] OR catfishes[tiab] OR cetacea[tiab] OR barbus[tiab] OR cygnus[tiab] OR ficedula[tiab] OR chamois[tiab] OR colubridae[tiab] OR perches[tiab] OR coelacanth[tiab] OR fitch[tiab] OR urodela[tiab] OR cynops[tiab] OR martes[tiab] OR halichoerus[tiab] OR aix[tiab] OR salmonidae[tiab] OR leuciscus[tiab] OR magpie[tiab] OR magpies[tiab] OR silurus[tiab] OR whiting[tiab] OR whitings[tiab] OR anseriformes[tiab] OR colinus[tiab] OR rhea[tiab] OR chlorocebus[tiab] OR octodon[tiab] OR acinonyx[tiab] OR mouflon[tiab] OR mouflons[tiab] OR ibex[tiab] OR tetraodon[tiab] OR bufonidae[tiab] OR equidae[tiab] OR jackal[tiab] OR cephalopoda[tiab] OR dendroaspis[tiab] OR glama[tiab] OR muskrat[tiab] OR muskrats[tiab] OR sable[tiab] OR sables[tiab] OR wildebeest[tiab] OR streptopelia[tiab] OR albifrons[tiab] OR vespertilionidae[tiab] OR woodpecker[tiab] OR woodpeckers[tiab] OR muntjac[tiab] OR muntjacs[tiab] OR archosaur[tiab] OR branta[tiab] OR cricetulus[tiab] OR megalobrama[tiab] OR poeciliidae[tiab] OR desmodus[tiab] OR snakehead[tiab] OR snakeheads[tiab] OR tench[tiab] OR teal[tiab] OR teals[tiab] OR bandicoot[tiab] OR bandicoots[tiab] OR apteronotus[tiab] OR phyllostomidae[tiab] OR crocidura[tiab] OR buzzard[tiab] OR buzzards[tiab] OR larimichthys[tiab] OR cercocebus[tiab] OR pipistrellus[tiab] OR erithacus[tiab] OR impala[tiab] OR impalas[tiab] OR rousettus[tiab] OR haddock[tiab] OR haddocks[tiab] OR tinca[tiab] OR ratite[tiab] OR calidris[tiab] OR cynoglossus[tiab] OR hypophthalmichthys[tiab] OR bullock[tiab] OR bullocks[tiab] OR dromedaries[tiab] OR alectoris[tiab] OR filly[tiab] OR salamandra[tiab] OR cingulata[tiab] OR bitis[tiab] OR grus[tiab] OR ammodytes[tiab] OR macaw[tiab] OR macaws[tiab] OR hypoleuca[tiab] OR sapajus[tiab] OR cyprinodontiformes[tiab] OR hippopotamus[tiab] OR pelophylax[tiab] OR capybara[tiab] OR capybaras[tiab] OR weasel[tiab] OR weasels[tiab] OR cairina[tiab] OR cynomys[tiab] OR lutra[tiab] OR cockatoo[tiab] OR cockatoos[tiab] OR lachesis[tiab] OR lagomorpha[tiab] OR rupicapra[tiab] OR daboia[tiab] OR orang utan[tiab] OR orang utans[tiab] OR platyrrhini[tiab] OR charadriiformes[tiab] OR micrurus[tiab] OR psittaciformes[tiab] OR spalax[tiab] OR loris[tiab] OR mustelidae[tiab] OR sylvilagus[tiab] OR vitticeps[tiab] OR cockatiel[tiab] OR mustelus[tiab] OR cottus[tiab] OR erythrocebus[tiab] OR dipodomys[tiab] OR platessa[tiab] OR callicebus[tiab] OR loricariidae[tiab] OR catostomus[tiab] OR cuneata[tiab] OR cyanistes[tiab] OR cyprinodon[tiab] OR sigmodontinae[tiab] OR elasmobranchii[tiab] OR trichechus[tiab] OR sauropsid[tiab] OR xenarthra[tiab] OR dormouse[tiab] OR perissodactyla[tiab] OR nautilus[tiab] OR cirrhinus[tiab] OR gulo[tiab] OR tragelaphus[tiab] OR merula[tiab] OR numida[tiab] OR sciaenidae[tiab] OR cerastes[tiab] OR sciuridae[tiab] OR gibbosus[tiab] OR octopuses[tiab] OR eland[tiab] OR elands[tiab] OR phyllomedusa[tiab] OR pogona[tiab] OR walrus[tiab] OR agamidae[tiab] OR leptodactylidae[tiab] OR ridibundus[tiab] OR leontopithecus[tiab] OR anteater[tiab] OR anteaters[tiab] OR pelodiscus[tiab] OR cebidae[tiab] OR columbianus[tiab] OR pelteobagrus fulvidraco[tiab] OR hominoidea[tiab] OR mandrillus[tiab] OR zonotrichia leucophrys[tiab] OR agama[tiab] OR gobiocypris[tiab] OR bearded dragon[tiab] OR bearded dragons[tiab] OR sarotherodon[tiab] OR talpa[tiab] OR discoglossus[tiab] OR hagfishes[tiab] OR sphenodon[tiab] OR gudgeon[tiab] OR amphiuma[tiab] OR aythya[tiab] OR tenrec[tiab] OR tenrec[tiab] OR hominidae[tiab] OR risoria[tiab] OR salamandridae[tiab] OR camelidae[tiab] OR columbiformes[tiab] OR latimeria[tiab] OR plover[tiab] OR plovers[tiab] OR afrotheria[tiab] OR falco sparverius[tiab] OR polecat[tiab] OR polecats[tiab] OR crotalinae[tiab] OR salvadora[tiab] OR tarsier[tiab] OR lucioperca[tiab] OR anchovies[tiab] OR lungfishes[tiab] OR terrapin[tiab] OR dromaius novaehollandiae[tiab] OR lateolabrax[tiab] OR eigenmannia[tiab] OR pelamis[tiab] OR theropithecus[tiab] OR murinae[tiab] OR gander[tiab] OR gymnotus[tiab] OR pseudacris[tiab] OR gymnophiona[tiab] OR gymnotiformes[tiab] OR laticauda[tiab] OR falconiformes[tiab] OR dugong[tiab] OR dugongs[tiab] OR pintail[tiab] OR pintails[tiab] OR rook[tiab] OR rooks[tiab] OR lasiurus[tiab] OR catshark[tiab] OR catsharks[tiab] OR micropogonias[tiab] OR red junglefowl[tiab] OR paddlefish[tiab] OR ophiophagus[tiab] OR hollandicus[tiab] OR nymphicus[tiab] OR pimelodidae[tiab] OR aepyceros[tiab] OR cobitidae[tiab] OR strigiformes[tiab] OR cobitis[tiab] OR dormice[tiab] OR alytes[tiab] OR calloselasma[tiab] OR guanaco[tiab] OR phasianidae[tiab] OR round goby[tiab] OR trichogaster[tiab] OR catarrhini[tiab] OR eelpout[tiab] OR eelpouts[tiab] OR galaxias[tiab] OR gaur[tiab] OR pungitius[tiab] OR suslik[tiab] OR susliks[tiab] OR flatfishes[tiab] OR percidae[tiab] OR caprinae[tiab] OR todarodes[tiab] OR osmerus[tiab] OR ameiurus[tiab] OR anthropoidea[tiab] OR castor canadensis[tiab] OR pouting[tiab] OR poutings[tiab] OR tetraodontiformes[tiab] OR arvicolinae[tiab] OR siamang[tiab] OR siamangs[tiab] OR castor fiber[tiab] OR nomascus[tiab] OR red knot[tiab] OR red knots[tiab] OR syngnathidae[tiab] OR iguanidae[tiab] OR eretmochelys[tiab] OR ursidae[tiab] OR callimico[tiab] OR columbidae[tiab] OR microhylidae[tiab] OR anaxyrus[tiab] OR menidia[tiab] OR pipistrelle[tiab] OR greylag[tiab] OR pipidae[tiab] OR scandentia[tiab] OR bowfin[tiab] OR bowfins[tiab] OR dendrobatidae[tiab] OR zenaida[tiab] OR bushbaby[tiab] OR harrier[tiab] OR harriers[tiab] OR macropodidae[tiab] OR pygerythrus[tiab] OR clupeidae[tiab] OR odorrana[tiab] OR corvidae[tiab] OR jerboa[tiab] OR jerboas[tiab] OR canutus[tiab] OR hylobatidae[tiab] OR clupeiformes[tiab] OR great cormorant[tiab] OR great cormorants[tiab] OR scorpaeniformes[tiab] OR chondrostean[tiab] OR garfish[tiab] OR proboscidea[tiab] OR psetta[tiab] OR diapsid[tiab] OR serotinus[tiab] OR tetrao[tiab] OR walruses[tiab] OR carcharhiniformes[tiab] OR leucoraja[tiab] OR pumpkinseed[tiab] OR dosidicus[tiab] OR acipenseriformes[tiab] OR daubentonii[tiab] OR emberizidae[tiab] OR gadiformes[tiab] OR hyraxes[tiab] OR stizostedion[tiab] OR wolverine[tiab] OR wolverines[tiab] OR lissotriton[tiab] OR acanthurus[tiab] OR centrarchidae[tiab] OR gloydius[tiab] OR laurasiatheria[tiab] OR limosa[tiab] OR psittacula[tiab] OR leporidae[tiab] OR proteidae[tiab] OR zander[tiab] OR zanders[tiab] OR arapaima[tiab] OR bagridae[tiab] OR cyprinodontidae[tiab] OR mithun[tiab] OR pandion[tiab] OR jackdaw[tiab] OR jackdaws[tiab] OR procyonidae[tiab] OR carus[tiab] OR jaculus[tiab] OR salmoniformes[tiab] OR common sole[tiab] OR common soles[tiab] OR protobothrops[tiab] OR calamita[tiab] OR brachyteles[tiab] OR trionyx[tiab] OR turdidae[tiab] OR boidae[tiab] OR luscinia[tiab] OR pugnax[tiab] OR euarchontoglires[tiab] OR saithe[tiab] OR saithes[tiab] OR symphalangus[tiab] OR aardvark[tiab] OR aardvarks[tiab] OR oystercatcher[tiab] OR oystercatchers[tiab] OR arius[tiab] OR corydoras[tiab] OR poacher[tiab] OR poachers[tiab] OR aurochs[tiab] OR cebuella[tiab] OR crecca[tiab] OR lemuridae[tiab] OR sirenia[tiab] OR lemmus[tiab] OR perdix[tiab] OR glires[tiab] OR lepidosaur[tiab] OR muskox[tiab] OR deinagkistrodon[tiab] OR pholidota[tiab] OR holocephali[tiab] OR cercopithecinae[tiab] OR clariidae[tiab] OR agapornis[tiab] OR doryteuthis[tiab] OR tyrannidae[tiab] OR dicroglossidae[tiab] OR godwit[tiab] OR godwits[tiab] OR monedula[tiab] OR pongidae[tiab] OR atheriniformes[tiab] OR colobinae[tiab] OR lophocebus[tiab] OR atelidae[tiab] OR cottidae[tiab] OR leucopsis[tiab] OR acanthuridae[tiab] OR didelphimorphia[tiab] OR elver[tiab] OR elvers[tiab] OR lapponica[tiab] OR dermoptera[tiab] OR european hake[tiab] OR european hakes[tiab] OR gerbillinae[tiab] OR banteng[tiab] OR hartebeest[tiab] OR hartebeests[tiab] OR hogget[tiab] OR haematopus[tiab] OR anguis fragilis[tiab] OR grey heron[tiab] OR grey herons[tiab] OR blue whiting[tiab] OR blue whitings[tiab] OR furnariidae[tiab] OR macrovipera[tiab] OR esocidae[tiab] OR lapwing[tiab] OR lapwings[tiab] OR mylopharyngodon[tiab] OR wallabia[tiab] OR beloniformes[tiab] OR potoroo[tiab] OR potoroos[tiab] OR athene noctua[tiab] OR pleuronectidae[tiab] OR bushbabies[tiab] OR muscicapidae[tiab] OR alligatoridae[tiab] OR fuligula[tiab] OR bush baby[tiab] OR guineafowl[tiab] OR spoonbill[tiab] OR spoonbills[tiab] OR viverridae[tiab] OR catostomidae[tiab] OR zebrafishes[tiab] OR ibexes[tiab] OR vendace[tiab] OR estrildidae[tiab] OR monotremata[tiab] OR sepiella[tiab] OR ambystomatidae[tiab] OR shelduck[tiab] OR shelducks[tiab] OR treeshrew[tiab] OR treeshrews[tiab] OR hoplobatrachus[tiab] OR pochard[tiab] OR hoolock[tiab] OR hoolocks[tiab] OR lynxes[tiab] OR antilope[tiab] OR antilopes[tiab] OR blackbuck[tiab] OR blackbucks[tiab] OR cricetinae[tiab] OR paramisgurnus[tiab] OR skylark[tiab] OR skylarks[tiab] OR soleidae[tiab] OR allobates[tiab] OR northern wheatear[tiab] OR northern wheatears[tiab] OR pitheciidae[tiab] OR takin[tiab] OR theria[tiab] OR vanellus[tiab] OR galaxiidae[tiab] OR lorisidae[tiab] OR ostralegus[tiab] OR palaeognathae[tiab] OR stone loach[tiab] OR alauda[tiab] OR callitrichinae[tiab] OR caniformia[tiab] OR duttaphrynus[tiab] OR ictaluridae[tiab] OR osteoglossiformes[tiab] OR poultries[tiab] OR curema[tiab] OR ruddy turnstone[tiab] OR ruddy turnstones[tiab] OR sheatfish[tiab] OR sunfishes[tiab] OR centropomidae[tiab] OR hemachatus[tiab] OR platalea[tiab] OR thamnophilidae[tiab] OR song thrush[tiab] OR atherinopsidae[tiab] OR siluridae[tiab] OR tadorna[tiab] OR chroicocephalus[tiab] OR ermine[tiab] OR ermines[tiab] OR gavialis[tiab] OR ruff[tiab] OR tupaiidae[tiab] OR diprotodontia[tiab] OR hyaenidae[tiab] OR antilopinae[tiab] ORcrocodylidae[tiab] OR herpestidae[tiab] OR hippopotamidae[tiab] OR northern shoveler[tiab] OR round gobies[tiab] OR cheirogaleidae[tiab] OR indriidae[tiab] OR fundulidae[tiab] OR pythonidae[tiab] OR rhynchocephalia[tiab] OR anodorhynchus[tiab] OR red-backed shrike[tiab] OR red-backed shrikes[tiab] OR triakidae[tiab] OR phalangeridae[tiab] OR aoudad[tiab] OR boreoeutheria[tiab] OR eurasian jay[tiab] OR eurasian jays[tiab] OR feliformia[tiab] OR haplorhini[tiab] OR osteoglossidae[tiab] OR paenungulata[tiab] OR struthioniformes[tiab] OR ferina[tiab] OR sanderling[tiab] OR sanderlings[tiab] OR spheniscidae[tiab] OR cuttlefishes[tiab] OR cygnet[tiab] OR dasycneme[tiab] OR gadwall[tiab] OR gadwalls[tiab] OR pelobates fuscus[tiab] OR wryneck[tiab] OR wrynecks[tiab] OR afrosoricida[tiab] OR culaea[tiab] OR dover sole[tiab] OR dover soles[tiab] OR paralichthyidae[tiab] OR passeridae[tiab] OR osteolaemus[tiab] OR song thrushes[tiab OR bluethroat[tiab] OR bluethroats[tiab] OR hydrophiidae[tiab] OR megrim[tiab] OR mephitidae[tiab] OR strepsirhini[tiab] OR tomistoma[tiab] OR epidalea[tiab] OR osmeriformes[tiab] OR bush babies[tiab] OR tarsiiform[tiab] OR atelinae[tiab] OR bufotes[tiab] OR eurasian coot[tiab] OR eurasian coots[tiab] OR galagidae[tiab] OR geopelia[tiab] OR philomachus[tiab] OR tubulidentata[tiab] OR bombinatoridae[tiab] OR pelobatidae[tiab] OR tachysurus[tiab] OR ailuridae[tiab] OR woodlark[tiab] OR woodlarks[tiab] OR alcelaphinae[tiab] OR redshank[tiab] OR redshanks[tiab] OR salientia[tiab] OR sand smelt[tiab] OR sand smelts[tiab] OR woodmice[tiab] OR woodmouse[tiab] OR dasyproctidae[tiab] OR eurasian wigeon[tiab] OR Eurasian wigeons[tiab] OR garganey[tiab] OR garganeys[tiab] OR lemon sole[tiab] OR lemon soles[tiab] OR common dab[tiab] OR common dabs[tiab] OR graylag[tiab] OR graylags[tiab] OR leucorodia[tiab] OR osphronemidae[tiab] OR bewickii[tiab] OR common moorhen[tiab] OR common moorhens[tiab] OR decapodiformes[tiab] OR gobbler[tiab] OR gobblers[tiab] OR odontophoridae[tiab] OR paddlefishes[tiab] OR eutheria[tiab] OR salmonine[tiab] OR esociformes[tiab] OR Eurasian woodcock[tiab] OR eurasian woodcocks[tiab] OR european smelt[tiab] OR european smelts[tiab] OR goldfishes[tiab] OR tenches[tiab] OR tyranni[tiab] OR common chaffinch[tiab] OR common chaffinchs[tiab] OR common redstart[tiab] OR common redstarts[tiab] OR common roach[tiab] OR common roachs[tiab] OR great knot[tiab] OR great knots[tiab] OR potoroidae[tiab] OR alytidae[tiab] OR coregonine[tiab] OR dipteral[tiab] OR leveret[tiab] OR poeciliopsis gracilis[tiab] OR amphiumidae[tiab] OR batrachoidiformes[tiab] OR bighead goby[tiab] OR heteropneustidae[tiab] OR lullula[tiab] OR norway pout[tiab] OR norway pouts[tiab] OR sipunculida[tiab] OR dogfishes[tiab] OR sebastidae[tiab] OR tarsiidae[tiab] OR alethinophidia[tiab] OR common nase[tiab] OR common nases[tiab] OR common sandpiper[tiab] OR common sandpipers[tiab] OR eurasian blackcap[tiab] OR eurasian blackcaps[tiab] OR pterocnemia[tiab] OR syngnathiformes[tiab] OR common chaffinches[tiab] OR eupleridae[tiab] OR octopodiformes[tiab] OR phascolarctidae[tiab] OR scophthalmidae[tiab] OR starry smooth-hound[tiab] OR starry smooth-hounds[tiab] OR whitefishes[tiab] OR cuniculidae[tiab] OR European sprat[tiab] OR european sprats[tiab] OR rosy bitterling[tiab] OR rosy bitterlings[tiab] OR common dace[tiab] OR common daces[tiab] OR lesser weever[tiab] OR lesser weevers[tiab] OR scaldfish[tiab] OR water rail[tiab] OR water rails[tiab] OR alouattinae[tiab] OR centrarchiformes[tiab] OR common whitethroat[tiab] OR common whitethroats[tiab] OR gavialidae[tiab] OR grey gurnard[tiab] OR grey gurnards[tiab] OR lateolabracidae[tiab] OR rheiformes[tiab] OR tub gurnard[tiab] OR tub gurnards[tiab] OR common chiffchaff[tiab] OR common chiffchaffs[tiab] OR garfishes[tiab] OR lesser whitethroat[tiab] OR lesser whitethroats[tiab] OR myoxidae[tiab] OR seabasses[tiab] OR spariformes[tiab] OR umbridae[tiab] OR yellow boxfish[tiab] OR anabantiformes[tiab] OR aotidae[tiab] OR common bleak[tiab] OR common bleaks[tiab] OR common rudd[tiab] OR common rudds[tiab] OR greater pipefish[tiab] OR hapale[tiab] OR nandiniidae[tiab] OR stone loaches[tiab] OR whinchat[tiab] OR whinchats[tiab] OR acanthuriformes[tiab] OR brotula barbata[tiab] OR common ling[tiab] OR common lings[tiab] OR common roaches[tiab] OR cottonrat[tiab] OR cottonrats[tiab] OR douroucoulis[tiab] OR dromaiidae[tiab] OR fitches[tiab] OR fitchew[tiab] OR galaxiiformes[tiab] OR laprine[tiab] OR saimiriinae[tiab] OR solenette[tiab] OR tarsii[tiab] OR tompot blenny[tiab] OR common dragonet[tiab] OR common dragonets[tiab] OR longspined bullhead[tiab] OR longspined bullheads[tiab] OR monotremate[tiab] OR monotremates[tiab] OR pempheriformes[tiab] OR perdicinae[tiab] OR presbytini[tiab] OR smegmamorpha[tiab] OR bighead gobies[tiab] OR carangaria incertae sedis[tiab] OR coiidae[tiab] OR fivebeard rockling[tiab] OR foulmart[tiab] OR foumart[tiab] OR grasskeet[tiab] OR greater pipefishes[tiab] OR ibices[tiab] OR millionfish[tiab] OR muguliformes[tiab] OR Norwegian topknot[tiab] OR peewit[tiab] OR red sea sailfin tang[tiab] OR rupicapras[tiab] OR sheatfishes[tiab] OR tompot blennies[tiab] OR twait shad[tiab] OR yellow boxfishes[tiab]) NOT medline[sb])

**Reference**

van der Mierden S, Hooijmans CR, Tillema AH, Rehn S, Bleich A, Leenaars CH. Laboratory animals search filter for different literature databases: PubMed, Embase, Web of Science and PsycINFO. Lab Anim. 2021 Sep 24:236772211045485. doi: 10.1177/00236772211045485. Epub ahead of print. PMID: 34559023.

# Supplementary material 3 – EMBASE Search

exp isoflurane/ OR (isofluran* OR isofluorane OR isoba OR isofor OR isoforine OR isoflo OR isorrane OR isorane OR sofloran OR forthane OR forane OR forene OR aerrane OR terrel OR 1 chloro 2,2,2 trifluoroethyl difluoromethyl ether).ti,ab,kf. OR (26675-46-7).ti,ab,kf,rn.

AND

(exp animal experiment/ OR exp animal model/ OR exp experimental animal/ OR exp transgenic animal/ OR exp male animal/ OR exp female animal/ OR exp juvenile animal/ OR animal/ OR chordata/ OR vertebrate/ OR tetrapod/ OR exp fish/ OR amniote/ OR exp amphibia/ OR mammal/ OR exp reptile/ OR exp sauropsid/ OR therian/ OR exp monotreme/ OR placental mammal/ OR exp marsupial/ OR Euarchontoglires/ OR exp Afrotheria/ OR exp Boreoeutheria/ OR exp Laurasiatheria/ OR exp Xenarthra/ OR primate/ OR exp Dermoptera/ OR exp Glires/ OR exp Scandentia/ OR Haplorhini/ OR exp prosimian/ OR simian/ OR exp tarsiiform/ OR Catarrhini/ OR exp Platyrrhini/ OR ape/ OR exp Cercopithecidae/ OR hominid/ OR exp hylobatidae/ OR exp chimpanzee/ OR exp gorilla/ OR exp orang utan/ OR exp cephalopod/) OR (rat OR rats OR animal OR animals OR mice OR "in vivo" OR mouse OR rabbit OR rabbits OR murine OR pig OR pigs OR dog OR dogs OR bovine OR fish OR vertebrate OR vertebrates OR cat OR cats OR rodent OR rodents OR mammal OR mammals OR

chicken OR chickens OR monkey OR monkeys OR sheep OR canine OR canines OR porcine OR cattle OR bird OR birds OR hamster OR hamsters OR primate OR primates OR cow OR cows OR chick OR horse OR horses OR avian OR avians OR calf OR swine OR swines OR xenopus OR turkeys OR bear OR bears OR frog OR frogs OR zebrafish OR goat OR goats OR equine OR calves OR poultry OR macaque OR macaques OR mole OR moles OR ovine OR lamb OR lambs OR fishes OR diptera OR amphibian OR amphibians OR snake OR snakes OR ruminant OR ruminants OR hen OR hens OR piglet OR piglets OR feline OR felines OR simian OR simians OR laevis OR trout OR trouts OR teleost OR teleosts OR salmon OR salmons OR seal OR seals OR bull OR bulls OR ewe OR ewes OR hedgehog OR hedgehogs OR macaca OR macacas OR proteus OR pigeon OR pigeons OR bat OR bats OR duck OR ducks OR chimpanzee OR chimpanzees OR baboon OR baboons OR deer OR deers OR rana OR ranas OR carp OR carps OR heifer OR swallow OR swallows OR lizard OR lizards OR canis OR sow OR sows OR

cynomolgus OR quail OR quails OR reptile OR reptiles OR turtle OR turtles OR buffalo OR gerbil OR gerbils OR boar OR boars OR squirrel OR squirrels OR oncorhynchus OR mus OR toad OR toads OR fowl OR fowls OR rerio OR danio OR ara OR aras OR musculus OR tadpole OR tadpoles OR mulatta OR salmo OR ram OR eagle OR eagles OR ferret OR ferrets OR goldfish OR catfish OR whale OR whales OR fox OR foxes OR ape OR apes OR elephant OR elephants OR bos OR marmoset OR

marmosets OR cod OR cods OR shark OR sharks OR wolf OR eel OR eels OR auratus OR rattus OR zebra OR zebras OR tilapia OR tilapias OR gilt OR camel OR camels OR squid OR gallus OR marsupial OR marsupials OR vole OR voles OR fascicularis OR ovis OR salmonid OR salmonids OR tiger OR tigers OR dolphin OR dolphins OR robin OR robins OR carpio OR opossum OR opossums OR cyprinus OR salamander OR salamanders OR felis OR mink OR minks OR swan OR swans OR norvegicus OR

bufo OR torpedo OR bass OR lamprey OR lampreys OR sus OR python OR pythons OR tetrapod OR tetrapods OR shrew OR shrews OR lion OR lions OR hog OR hogs OR songbird OR songbirds OR oreochromis OR starling OR starlings OR caprine OR carassius OR owl OR owls OR newt OR newts OR papio OR scrofa OR hare OR hares OR gorilla OR gorillas OR flounder OR flounders OR goose OR herring OR herrings OR therian OR buffaloes OR canary OR sparrow OR sparrows OR microtus OR

octopus OR troglodytes OR tuna OR amphibia OR chinchilla OR chinchillas OR ide OR oryzias OR cervus OR kangaroo OR kangaroos OR armadillo OR armadillos OR callithrix OR "pan troglodytes" OR saimiri OR cichlid OR cichlids OR donkey OR donkeys OR bream OR char OR chars OR finch OR raccoon OR raccoons OR bothrops OR anguilla OR perch OR cricetus OR seabird OR seabirds OR buck OR bucks OR naja OR coturnix OR salmonids OR geese OR minnow OR minnows OR raptor OR

raptors OR merione OR meriones OR rodentia OR elaphus OR amniote OR amniotes OR elasmobranch OR emu OR emus OR peromyscus OR hominid OR hominids OR bubalus OR crotalus OR gull OR gulls OR anas OR anura OR lemur OR lemurs OR crow OR crows OR camelus OR gibbon OR gibbons OR waterfowl OR parrot OR parrots OR eels OR cob OR stickleback OR

sticklebacks OR columba OR mesocricetus OR ambystoma OR raven OR ravens OR gadus OR penguin OR penguins OR orangutan OR orangutans OR sturgeon OR sturgeons OR cuniculus OR aves OR virginianus OR cephalopod OR cephalopods OR cebus OR sparus OR tortoise OR tortoises OR guttata OR morhua OR unguiculatus OR dogfish OR vulpes OR mallard OR mallards OR apodemus OR alligator OR alligators OR oryctolagus OR llama OR llamas OR reindeer OR mustela OR duckling

OR ducklings OR wolves OR sander OR amazona OR zebu OR badger OR badgers OR dove OR doves OR ictalurus OR capra OR capras OR equus OR camelid OR camelids OR poecilia OR mule OR mules OR perciformes OR salvelinus OR labrax OR cyprinidae OR ariidae OR crocodile OR crocodiles OR fundulus OR dicentrarchus OR clarias OR cercopithecus OR chiroptera OR alpaca OR alpacas OR pike OR pikes OR paralichthys OR puma OR pumas OR didelphis OR pisces OR macropus OR triturus OR bison OR bisons OR epinephelus OR gasterosteus OR panthera OR acipenser OR mackerel OR mackerels OR tamarin OR tamarins OR ostrich OR anolis OR vervet OR vervets OR wallaby OR glareolus OR beaver OR beavers OR dromedary OR catus OR killifish OR pimephales OR promelas OR aotus OR phoca OR panda OR pandas OR porpoise OR porpoises OR myotis OR yak OR yaks OR agkistrodon OR vipera OR otter OR otters OR turbot OR turbots OR squamate OR carnivora OR mullet OR mullets OR hawk OR hawks OR taeniopygia OR seahorse OR seahorses OR "poecilia reticulata" OR falcon OR falcons OR prosimian OR prosimians OR parus OR perca OR fingerling OR fingerlings OR antelope OR antelopes OR tupaia OR passeriformes OR sepia OR saguinus OR coyote OR coyotes OR pongo OR meleagris OR reptilia OR lepus OR psittacine OR hagfish OR warbler OR warblers OR "russell s viper" OR "russell s vipers" OR smolt OR smolts OR budgerigar OR sardine OR sardines OR cavia OR cavias OR hyla OR pleurodeles OR siluriformes OR "great tit" OR "great tits" OR guppy OR bonobo OR bonobos OR rutilus OR trichosurus OR muridae OR phodopus OR channa OR squalus OR lynx OR sturnus OR petromyzon OR vitulina OR monodelphis OR cuttlefish OR adder OR adders OR lepomis OR canaria OR gambusia OR guppies OR xiphophorus OR flatfish OR koala OR koalas OR labeo OR stingray OR stingrays OR chelonia OR lampetra OR spermophilus OR crocodilian OR "passer domesticus" OR sciurus OR artiodactyla OR ranidae OR corvus OR necturus OR

platypus OR canaries OR bovid OR lagopus OR trimeresurus OR gariepinus OR marten OR martens OR drosophilidae OR mugil OR sunfish OR porcellus OR cypriniformes OR alouatta OR scophthalmus OR anser OR electrophorus OR putorius OR iguana OR iguanas OR lama OR lamas OR takifugu OR circus OR eptesicus OR flycatcher OR galago OR galagos OR Trachemys OR lungfish OR characiformes OR shorebird OR shorebirds OR giraffe OR giraffes OR micropterus OR scyliorhinus OR cichlidae OR loligo OR porcupine OR porcupines OR chub OR chubs OR solea OR pleuronectes OR hylidae OR viperidae OR echis OR sorex OR anchovy OR lagomorph OR ostriches OR vulture OR vultures OR whitefish OR araneus OR jird OR jirds OR tern OR esox OR drake OR drakes OR elapidae OR gallopavo OR chordata OR myodes OR caretta OR serinus OR grouse OR misgurnus OR meles OR blackbird OR blackbirds OR coregonus OR bobwhite OR bobwhites OR heteropneustes OR mammoth OR mammoths OR turdus OR rhinella OR ateles OR characidae OR clupea OR bungarus OR brill OR "struthio camelus" OR sloth OR sloths OR pteropus OR sculpin OR anthropoids OR pollock OR pollocks OR morone OR "pan paniscus" OR litoria OR chipmunk OR chipmunks OR balaenoptera OR marmota OR melopsittacus OR hyrax OR lemming OR lemmings OR halibut OR hylobates OR lates OR caiman OR caimans OR sigmodon OR stenella OR barbel OR barbels OR sterna OR parakeet OR parakeets OR phocoena OR leptodactylus OR canidae OR buteo OR harengus OR gopher OR gophers OR marmot OR marmots OR gosling OR goslings OR platichthys OR gar OR gars OR sebastes OR marsupialia OR notophthalmus OR gazelle OR gazelles OR insectivora OR paridae OR felidae OR russula OR galliformes OR bombina OR colobus OR echidna OR echidnas OR seabass OR syncerus OR plaice OR "blue tit" OR "blue tits" OR pagrus OR catfishes OR cetacea OR barbus OR cygnus OR ficedula OR chamois OR colubridae OR perches OR coelacanth OR fitch OR urodela OR cynops OR martes OR halichoerus OR aix OR salmonidae OR leuciscus OR magpie OR magpies OR silurus OR whiting OR whitings OR anseriformes OR colinus OR rhea OR chlorocebus OR octodon OR acinonyx OR mouflon OR mouflons OR ibex OR tetraodon OR bufonidae OR equidae OR jackal OR ephalopoda OR dendroaspis OR glama OR muskrat OR muskrats OR sable OR sables OR wildebeest OR treptopelia OR albifrons OR vespertilionidae OR woodpecker OR woodpeckers OR muntjac OR muntjacs OR archosaur OR branta OR cricetulus OR megalobrama OR poeciliidae OR desmodus OR snakehead OR snakeheads OR tench OR teal OR teals OR bandicoot OR bandicoots OR apteronotus OR phyllostomidae OR crocidura OR buzzard OR buzzards OR larimichthys OR cercocebus OR pipistrellus OR erithacus OR impala OR impalas OR rousettus OR haddock OR haddocks OR

tinca OR ratite OR calidris OR cynoglossus OR hypophthalmichthys OR bullock OR bullocks OR dromedaries OR alectoris OR filly OR salamandra OR cingulata OR bitis OR grus OR ammodytes OR macaw OR macaws OR hypoleuca OR sapajus OR cyprinodontiformes OR hippopotamus OR elophylax OR capybara OR capybaras OR weasel OR weasels OR cairina OR cynomys OR lutra OR cockatoo OR cockatoos OR lachesis OR lagomorpha OR rupicapra OR daboia OR "orang utan" OR

"orang utans" OR platyrrhini OR charadriiformes OR micrurus OR psittaciformes OR spalax OR loris OR mustelidae OR sylvilagus OR vitticeps OR cockatiel OR mustelus OR cottus OR erythrocebus OR dipodomys OR platessa OR callicebus OR loricariidae OR catostomus OR cuneata OR cyanistes OR cyprinodon OR sigmodontinae OR elasmobranchii OR trichechus OR sauropsid OR xenarthra OR dormouse OR perissodactyla OR nautilus OR cirrhinus OR gulo OR gulos OR tragelaphus OR

merula OR numida OR sciaenidae OR cerastes OR sciuridae OR gibbosus OR octopuses OR eland OR elands OR phyllomedusa OR pogona OR walrus OR agamidae OR leptodactylidae OR ridibundus OR leontopithecus OR anteater OR anteaters OR pelodiscus OR cebidae OR columbianus OR "pelteobagrus fulvidraco" OR hominoidea OR mandrillus OR "zonotrichia leucophrys" OR agama OR gobiocypris OR "bearded dragon" OR "bearded dragons" OR sarotherodon OR talpa OR discoglossus OR hagfishes OR sphenodon OR gudgeon OR amphiuma OR aythya OR tenrec OR tenrec OR hominidae OR risoria OR salamandridae OR camelidae OR columbiformes OR latimeria OR plover OR plovers OR afrotheria OR "falco sparverius" OR polecat OR polecats OR crotalinae OR salvadora OR tarsier OR lucioperca OR anchovies OR lungfishes OR terrapin OR "dromaius novaehollandiae" OR lateolabrax OR eigenmannia OR pelamis OR theropithecus OR murinae OR gander OR gymnotus OR pseudacris OR gymnophiona OR gymnotiformes OR laticauda OR falconiformes OR dugong OR

dugongs OR pintail OR pintails OR rook OR rooks OR lasiurus OR catshark OR catsharks OR micropogonias OR "red junglefowl" OR paddlefish OR ophiophagus OR hollandicus OR nymphicus OR pimelodidae OR aepyceros OR cobitidae OR strigiformes OR cobitis OR dormice OR alytes OR calloselasma OR guanaco OR guanacos OR phasianidae OR "round goby" OR trichogaster OR catarrhini OR eelpout OR eelpouts OR galaxias OR gaur OR pungitius OR suslik OR susliks OR flatfishes OR percidae OR caprinae OR todarodes OR osmerus OR ameiurus OR anthropoidea OR "castor canadensis" OR pouting OR poutings OR tetraodontiformes OR arvicolinae OR siamang OR siamangs OR "castor fiber" OR nomascus OR "red knot" OR "red knots" OR syngnathidae OR iguanidae OR eretmochelys OR ursidae OR callimico OR columbidae OR microhylidae OR

anaxyrus OR menidia OR pipistrelle OR greylag OR pipidae OR scandentia OR bowfin OR bowfins OR dendrobatidae OR zenaida OR bushbaby OR harrier OR harriers OR macropodidae OR pygerythrus OR clupeidae OR odorrana OR corvidae OR jerboa OR jerboas OR canutus OR hylobatidae OR clupeiformes OR "great cormorant" OR "great cormorants" OR scorpaeniformes OR chondrostean OR garfish OR proboscidea OR psetta OR diapsid OR serotinus OR tetrao OR walruses OR

carcharhiniformes OR leucoraja OR pumpkinseed OR dosidicus OR acipenseriformes OR daubentonii OR emberizidae OR gadiformes OR hyraxes OR stizostedion OR wolverine OR wolverines OR lissotriton OR acanthurus OR centrarchidae OR gloydius OR laurasiatheria OR limosa OR psittacula OR leporidae OR proteidae OR zander OR zanders OR arapaima OR bagridae OR cyprinodontidae OR mithun OR pandion OR jackdaw OR jackdaws OR procyonidae OR carus OR jaculus OR

salmoniformes OR "common sole" OR "common soles" OR protobothrops OR calamita OR brachyteles OR trionyx OR turdidae OR boidae OR luscinia OR pugnax OR euarchontoglires OR saithe OR saithes OR symphalangus OR aardvark OR aardvarks OR oystercatcher OR oystercatchers OR arius OR corydoras OR poacher OR poachers OR aurochs OR cebuella OR crecca OR lemuridae OR sirenia OR lemmus OR perdix OR glires OR lepidosaur OR muskox OR deinagkistrodon OR pholidota

OR holocephali OR cercopithecinae OR clariidae OR agapornis OR doryteuthis OR tyrannidae OR dicroglossidae OR godwit OR godwits OR monedula OR pongidae OR atheriniformes OR colobinae OR lophocebus OR atelidae OR cottidae OR leucopsis OR acanthuridae OR didelphimorphia OR elver OR elvers OR lapponica OR dermoptera OR "european hake" OR "european hakes" OR gerbillinae OR banteng OR hartebeest OR hartebeests OR hogget OR haematopus OR "anguis fragilis" OR "grey heron" OR "grey herons" OR "blue whiting" OR "blue whitings" OR furnariidae OR macrovipera OR esocidae OR lapwing OR lapwings OR mylopharyngodon OR wallabia OR beloniformes OR potoroo OR potoroos OR "athene noctua" OR pleuronectidae OR bushbabies OR muscicapidae OR alligatoridae OR fuligula OR "bush baby" OR guineafowl OR spoonbill OR spoonbills OR viverridae OR catostomidae OR zebrafishes OR ibexes OR vendace OR estrildidae OR monotremata OR sepiella OR ambystomatidae OR shelduck OR shelducks OR treeshrew OR treeshrews OR hoplobatrachus OR pochard OR hoolock OR hoolocks OR lynxes OR antilope OR antilopes OR blackbuck OR blackbucks OR cricetinae OR paramisgurnus OR skylark OR skylarks OR soleidae OR allobates OR "northern wheatear" OR "northern wheatears" OR pitheciidae OR takin OR theria OR vanellus OR galaxiidae OR lorisidae OR ostralegus OR palaeognathae OR "stone loach" OR alauda OR callitrichinae OR caniformia OR duttaphrynus OR ictaluridae OR osteoglossiformes OR poultries OR curema OR "ruddy turnstone" OR "ruddy turnstones" OR sheatfish OR sunfishes OR centropomidae OR hemachatus OR platalea OR thamnophilidae OR "song thrush" OR atherinopsidae OR siluridae OR tadorna OR chroicocephalus OR ermine OR ermines OR gavialis OR ruff OR tupaiidae OR diprotodontia OR hyaenidae OR antilopinae OR crocodylidae OR herpestidae OR hippopotamidae OR "northern shoveler" OR "round gobies" OR cheirogaleidae OR indriidae OR fundulidae OR pythonidae OR rhynchocephalia OR anodorhynchus OR "red-backed shrike" OR "red-backed shrikes" OR triakidae OR phalangeridae OR aoudad OR boreoeutheria OR "eurasian jay" OR "eurasian jays" OR feliformia OR haplorhini OR osteoglossidae OR paenungulata OR struthioniformes OR ferina OR sanderling OR sanderlings OR spheniscidae OR cuttlefishes OR cygnet OR dasycneme OR gadwall OR gadwalls OR "pelobates fuscus" OR wryneck OR wrynecks OR afrosoricida OR culaea OR "dover sole" OR "dover soles" OR paralichthyidae OR passeridae OR osteolaemus OR "song thrushes" OR bluethroat OR bluethroats OR hydrophiidae OR megrim OR mephitidae OR strepsirhini OR tomistoma OR epidalea OR osmeriformes OR "bush babies" OR tarsiiform OR atelinae OR bufotes OR "eurasian coot" OR "eurasian coots" OR galagidae OR geopelia OR philomachus OR tubulidentata OR bombinatoridae OR pelobatidae OR tachysurus OR ailuridae OR woodlark OR woodlarks OR alcelaphinae OR redshank OR redshanks OR salientia OR "sand smelt" OR "sand smelts" OR woodmice OR woodmouse OR dasyproctidae OR "eurasian wigeon" OR "eurasian wigeons" OR garganey OR garganeys OR "lemon sole" OR "lemon soles" OR "common dab" OR "common dabs" OR graylag OR graylags OR leucorodia OR osphronemidae OR bewickii OR "common moorhen" OR "common moorhens" OR decapodiformes OR gobbler OR gobblers OR odontophoridae OR paddlefishes OR eutheria OR salmonine OR esociformes OR "eurasian woodcock" OR "eurasian woodcocks" OR "european smelt" OR "european smelts" OR goldfishes OR tenches OR tyranni OR "common chaffinch" OR "common chaffinchs" OR "common redstart" OR "common redstarts" OR "common roach" OR "common roachs" OR "great knot" OR "great knots" OR potoroidae OR alytidae OR coregonine OR dipteral OR leveret OR "poeciliopsis gracilis" OR amphiumidae OR batrachoidiformes OR "bighead goby" OR heteropneustidae OR lullula OR "norway pout" OR "norway pouts" OR sipunculida OR dogfishes OR sebastidae OR tarsiidae OR alethinophidia OR "common nase" OR "common nases" OR "common sandpiper" OR "common sandpipers" OR "eurasian blackcap" OR "eurasian blackcaps" OR pterocnemia OR syngnathiformes OR "common chaffinches" OR eupleridae OR octopodiformes OR phascolarctidae OR scophthalmidae OR "starry smooth-hound" OR "starry smooth-hounds" OR whitefishes OR cuniculidae OR "european sprat" OR "european sprats" OR "rosy bitterling" OR "rosy bitterlings" OR "common dace" OR "common daces" OR "lesser weever" OR "lesser weevers" OR scaldfish OR "water rail" OR "water rails" OR alouattinae OR centrarchiformes OR "common whitethroat" OR "common whitethroats" OR gavialidae OR "grey gurnard" OR "grey gurnards" OR lateolabracidae OR rheiformes OR "tub gurnard" OR "tub gurnards" OR "common chiffchaff" OR "common chiffchaffs" OR garfishes OR "lesser whitethroat" OR "lesser whitethroats" OR myoxidae OR seabasses OR spariformes OR umbridae OR "yellow boxfish" OR anabantiformes OR aotidae OR "common bleak" OR "common bleaks" OR "common rudd" OR "common rudds" OR "greater pipefish" OR hapale OR nandiniidae OR "stone loaches" OR whinchat OR whinchats OR acanthuriformes OR "brotula barbata" OR "common ling" OR "common lings" OR "common roaches" OR cottonrat OR cottonrats OR douroucoulis OR dromaiidae OR fitches OR fitchew OR galaxiiformes OR laprine OR saimiriinae OR solenette OR tarsii OR "tompot blenny" OR "common dragonet" OR "common dragonets" OR "longspined bullhead" OR "longspined bullheads" OR monotremate OR monotremates OR pempheriformes OR perdicinae OR presbytini OR smegmamorpha OR "bighead gobies" OR "carangaria incertae sedis" OR coiidae OR "fivebeard rockling" OR foulmart OR foumart OR grasskeet OR "greater pipefishes" OR ibices OR millionfish OR muguliformes OR "norwegian topknot" OR peewit OR "red sea sailfin tang" OR rupicapras OR sheatfishes OR "tompot blennies" OR "twait shad" OR "yellow boxfishes").ti,ab,kw.

# Supplementary material 4 – Web of Science Search

TOPIC: (isofluran* OR isofluorane OR isoba OR isofor OR isoforine OR isoflo OR isorrane OR isorane OR sofloran OR forthane OR forane OR forene OR aerrane OR terrel OR 1 chloro 2,2,2 trifluoroethyl difluoromethyl ether OR 26675-46-7)

AND

TS=(rat OR rats OR animal OR animals OR mice OR "in vivo" OR mouse OR rabbit OR rabbits OR murine OR pig OR pigs OR dog OR dogs OR bovine OR fish OR vertebrate OR vertebrates OR cat OR cats OR rodent OR rodents OR mammal OR mammals OR chicken OR chickens OR monkey OR monkeys OR sheep OR canine OR canines OR porcine OR cattle OR bird OR birds OR hamster OR hamsters OR primate OR primates OR cow OR cows OR chick OR horse OR horses OR avian OR

avians OR calf OR swine OR swines OR xenopus OR turkeys OR bear OR bears OR frog OR frogs OR zebrafish OR goat OR goats OR equine OR calves OR poultry OR macaque OR macaques OR mole OR moles OR ovine OR lamb OR lambs OR fishes OR diptera OR amphibian OR amphibians OR snake OR snakes OR ruminant OR ruminants OR hen OR hens OR piglet OR piglets OR feline OR felines OR simian OR simians OR laevis OR trout OR trouts OR teleost OR teleosts OR salmon OR salmons OR seal OR seals OR bull OR bulls OR ewe OR ewes OR hedgehog OR hedgehogs OR macaca OR macacas OR proteus OR pigeon OR pigeons OR bat OR bats OR duck OR ducks OR chimpanzee OR chimpanzees OR baboon OR baboons OR deer OR rana OR ranas OR carp OR carps OR heifer OR swallow OR swallows OR lizard OR lizards OR canis OR sow OR sows OR cynomolgus OR quail OR quails OR reptile OR reptiles OR turtle OR turtles OR buffalo OR gerbil OR gerbils OR boar

OR boars OR squirrel OR squirrels OR oncorhynchus OR mus OR toad OR toads OR fowl OR fowls OR rerio OR danio OR ara OR aras OR musculus OR tadpole OR tadpoles OR mulatta OR salmo OR ram OR eagle OR eagles OR ferret OR ferrets OR goldfish OR catfish OR whale OR whales OR fox OR foxes OR ape OR apes OR elephant OR elephants OR bos OR marmoset OR marmosets OR cod OR cods OR shark OR sharks OR wolf OR eel OR eels OR auratus OR rattus OR zebra OR zebras OR

tilapia OR tilapias OR gilt OR camel OR camels OR squid OR gallus OR marsupial OR marsupials OR vole OR voles OR fascicularis OR ovis OR salmonid OR salmonids OR tiger OR tigers OR dolphin OR dolphins OR robin OR robins OR carpio OR opossum OR opossums OR cyprinus OR salamander OR salamanders OR felis OR mink OR minks OR swan OR swans OR norvegicus OR bufo OR torpedo OR bass OR lamprey OR lampreys OR sus OR python OR pythons OR tetrapod OR tetrapods OR shrew OR shrews OR lion OR lions OR hog OR hogs OR songbird OR songbirds OR oreochromis OR starling OR starlings OR caprine OR carassius OR owl OR owls OR newt OR newts OR papio OR scrofa OR hare OR hares OR gorilla OR gorillas OR flounder OR flounders OR goose OR herring OR herrings OR therian OR buffaloes OR canary OR sparrow OR sparrows OR microtus OR octopus OR troglodytes OR tuna OR amphibia OR chinchilla OR chinchillas OR ide OR oryzias OR cervus OR kangaroo OR kangaroos OR armadillo OR armadillos OR callithrix OR "pan troglodytes" OR saimiri OR cichlid OR cichlids OR

donkey OR donkeys OR bream OR char OR chars OR finch OR raccoon OR raccoons OR bothrops OR anguilla OR perch OR cricetus OR seabird OR seabirds OR buck OR bucks OR naja OR coturnix OR salmonids OR geese OR minnow OR minnows OR raptor OR raptors OR merione OR meriones OR rodentia OR elaphus OR amniote OR amniotes OR elasmobranch OR emu OR emus OR peromyscus OR hominid OR hominids OR bubalus OR crotalus OR gull OR gulls OR anas OR anura OR lemur OR

lemurs OR crow OR crows OR camelus OR gibbon OR gibbons OR waterfowl OR parrot OR parrots OR eels OR cob OR stickleback OR sticklebacks OR columba OR mesocricetus OR ambystoma OR raven OR ravens OR gadus OR penguin OR penguins OR orangutan OR orangutans OR sturgeon OR sturgeons OR cuniculus OR aves OR virginianus OR cephalopod OR cephalopods OR cebus OR sparus OR tortoise OR tortoises OR guttata OR morhua OR unguiculatus OR dogfish OR vulpes OR mallard OR mallards OR apodemus OR alligator OR alligators OR oryctolagus OR llama OR llamas OR reindeer OR mustela OR duckling OR ducklings OR wolves OR sander OR amazona OR zebu OR badger OR badgers OR dove OR doves OR ictalurus OR capra OR capras OR equus OR camelid OR camelids OR poecilia OR mule OR mules OR perciformes OR salvelinus OR labrax OR cyprinidae OR ariidae OR crocodile OR crocodiles OR fundulus OR dicentrarchus OR clarias OR cercopithecus OR chiroptera OR alpaca OR alpacas OR pike OR pikes OR paralichthys OR puma OR pumas OR didelphis OR pisces OR macropus OR triturus OR bison OR bisons OR epinephelus OR gasterosteus OR panthera OR acipenser OR mackerel OR mackerels OR tamarin OR tamarins OR ostrich OR anolis OR vervet OR vervets OR wallaby OR glareolus OR beaver OR beavers OR dromedary OR catus OR killifish OR pimephales OR promelas OR aotus OR phoca OR panda OR pandas OR porpoise OR porpoises OR myotis OR yak OR yaks OR agkistrodon OR vipera OR otter OR otters OR turbot OR turbots OR squamate OR carnivora OR mullet OR mullets OR hawk OR hawks OR taeniopygia OR seahorse OR seahorses OR "poecilia reticulata" OR falcon OR falcons OR prosimian OR prosimians OR parus OR perca OR fingerling OR fingerlings OR antelope OR antelopes OR tupaia OR passeriformes OR sepia OR saguinus OR coyote OR coyotes OR pongo OR meleagris OR reptilia OR lepus OR psittacine OR hagfish OR warbler OR warblers OR "russell s viper" OR "russell s vipers" OR smolt OR smolts OR budgerigar OR sardine OR sardines OR cavia OR cavias OR hyla OR pleurodeles OR siluriformes OR "great tit" OR "great tits" OR guppy OR bonobo OR bonobos OR rutilus OR trichosurus OR muridae OR phodopus OR channa OR squalus OR lynx OR sturnus OR petromyzon OR vitulina OR onodelphis OR cuttlefish OR adder OR adders OR lepomis OR canaria OR gambusia OR guppies OR xiphophorus OR flatfish OR koala OR koalas OR labeo OR stingray OR stingrays OR chelonia OR lampetra OR

spermophilus OR crocodilian OR "passer domesticus" OR sciurus OR artiodactyla OR ranidae OR corvus OR necturus OR platypus OR canaries OR bovid OR lagopus OR trimeresurus OR gariepinus OR marten OR martens OR drosophilidae OR mugil OR sunfish OR porcellus OR cypriniformes OR alouatta OR scophthalmus OR anser OR electrophorus OR putorius OR iguana OR iguanas OR lama OR lamas OR takifugu OR circus OR eptesicus OR flycatcher OR galago OR galagos OR trachemys

OR lungfish OR characiformes OR shorebird OR shorebirds OR giraffe OR giraffes OR micropterus OR scyliorhinus OR cichlidae OR loligo OR porcupine OR porcupines OR chub OR chubs OR solea OR pleuronectes OR hylidae OR viperidae OR echis OR sorex OR anchovy OR lagomorph OR ostriches OR vulture OR vultures OR whitefish OR araneus OR jird OR jirds OR tern OR esox OR drake OR drakes OR elapidae OR gallopavo OR chordata OR myodes OR caretta OR serinus OR grouse OR misgurnus OR meles OR blackbird OR blackbirds OR coregonus OR bobwhite OR bobwhites OR heteropneustes OR mammoth OR mammoths OR turdus OR rhinella OR ateles OR characidae OR clupea OR bungarus OR brill OR "struthio camelus" OR sloth OR sloths OR pteropus OR sculpin OR anthropoids OR pollock OR pollocks OR morone OR "pan paniscus" OR litoria OR chipmunk OR chipmunks OR balaenoptera OR marmota OR melopsittacus OR hyrax OR lemming OR lemmings OR halibut OR hylobates OR lates OR caiman OR caimans OR sigmodon OR stenella OR barbel OR barbels OR sterna OR parakeet OR parakeets OR phocoena OR leptodactylus OR canidae OR buteo OR harengus OR gopher OR gophers OR marmot OR marmots OR gosling OR goslings OR platichthys OR gar OR gars OR sebastes OR marsupialia OR notophthalmus OR gazelle OR gazelles OR insectivora OR paridae OR felidae OR russula OR galliformes OR bombina OR colobus OR echidna OR echidnas OR seabass OR syncerus OR plaice OR "blue tit" OR "blue tits" OR pagrus OR catfishes OR cetacea OR barbus OR cygnus OR ficedula OR chamois OR colubridae OR perches OR coelacanth OR fitch OR urodela OR cynops OR martes OR halichoerus OR aix OR salmonidae OR leuciscus OR magpie OR magpies OR silurus OR whiting OR whitings OR anseriformes OR colinus OR rhea OR chlorocebus OR octodon OR acinonyx OR mouflon OR mouflons OR ibex OR tetraodon OR bufonidae OR equidae OR jackal OR ephalopoda OR dendroaspis OR glama OR muskrat OR muskrats OR sable OR sables OR wildebeest OR streptopelia OR albifrons OR vespertilionidae OR woodpecker OR woodpeckers OR muntjac OR muntjacs OR archosaur OR branta OR cricetulus OR megalobrama OR poeciliidae OR desmodus OR snakehead OR snakeheads OR tench OR teal OR teals OR bandicoot OR bandicoots OR apteronotus OR phyllostomidae OR crocidura OR buzzard OR buzzards OR larimichthys OR cercocebus OR pipistrellus OR erithacus OR impala OR impalas OR rousettus OR haddock OR haddocks OR

tinca OR ratite OR calidris OR cynoglossus OR hypophthalmichthys OR bullock OR bullocks OR dromedaries OR alectoris OR filly OR salamandra OR cingulata OR bitis OR grus OR ammodytes OR macaw OR macaws OR hypoleuca OR sapajus OR cyprinodontiformes OR hippopotamus OR pelophylax OR capybara OR capybaras OR weasel OR weasels OR cairina OR cynomys OR lutra OR cockatoo OR cockatoos OR lachesis OR lagomorpha OR rupicapra OR daboia OR "orang utan" OR

"orang utans" OR platyrrhini OR charadriiformes OR micrurus OR psittaciformes OR spalax OR loris OR mustelidae OR sylvilagus OR vitticeps OR cockatiel OR mustelus OR cottus OR erythrocebus OR dipodomys OR platessa OR callicebus OR loricariidae OR catostomus OR cuneata OR cyanistes OR cyprinodon OR sigmodontinae OR elasmobranchii OR trichechus OR sauropsid OR xenarthra OR dormouse OR perissodactyla OR nautilus OR cirrhinus OR gulo OR gulos OR tragelaphus OR

merula OR numida OR sciaenidae OR cerastes OR sciuridae OR gibbosus OR octopuses OR eland OR elands OR phyllomedusa OR pogona OR walrus OR agamidae OR leptodactylidae OR ridibundus OR leontopithecus OR anteater OR anteaters OR pelodiscus OR cebidae OR columbianus OR "pelteobagrus fulvidraco" OR hominoidea OR mandrillus OR "zonotrichia leucophrys" OR agama OR gobiocypris OR "bearded dragon" OR "bearded dragons" OR sarotherodon OR talpa OR discoglossus OR hagfishes OR sphenodon OR gudgeon OR amphiuma OR aythya OR tenrec OR tenrec OR hominidae OR risoria OR salamandridae OR camelidae OR columbiformes OR latimeria OR plover OR plovers OR afrotheria OR "falco sparverius" OR polecat OR polecats OR crotalinae OR salvadora OR tarsier OR lucioperca OR anchovies OR lungfishes OR terrapin OR "dromaius novaehollandiae" OR lateolabrax OR eigenmannia OR pelamis OR theropithecus OR murinae OR gander OR gymnotus OR pseudacris OR gymnophiona OR gymnotiformes OR laticauda OR falconiformes OR dugong OR

dugongs OR pintail OR pintails OR rook OR rooks OR lasiurus OR catshark OR catsharks OR micropogonias OR "red junglefowl" OR paddlefish OR eutheria OR ophiophagus OR hollandicus OR nymphicus OR pimelodidae OR aepyceros OR cobitidae OR strigiformes OR cobitis OR dormice OR alytes OR calloselasma OR guanaco OR guanacos OR phasianidae OR "round goby" OR trichogaster OR catarrhini OR eelpout OR eelpouts OR galaxias OR gaur OR pungitius OR suslik OR susliks

OR flatfishes OR percidae OR caprinae OR todarodes OR osmerus OR ameiurus OR anthropoidea OR "castor canadensis" OR pouting OR poutings OR tetraodontiformes OR arvicolinae OR siamang OR siamangs OR "castor fiber" OR nomascus OR "red knot" OR "red knots" OR syngnathidae OR iguanidae OR eretmochelys OR ursidae OR callimico OR columbidae OR microhylidae OR anaxyrus OR menidia OR pipistrelle OR greylag OR pipidae OR scandentia OR bowfin OR bowfins OR

dendrobatidae OR zenaida OR bushbaby OR harrier OR harriers OR macropodidae OR pygerythrus OR clupeidae OR odorrana OR corvidae OR jerboa OR jerboas OR canutus OR hylobatidae OR clupeiformes OR "great cormorant" OR "great cormorants" OR scorpaeniformes OR chondrostean OR garfish OR proboscidea OR psetta OR diapsid OR serotinus OR tetrao OR walruses OR carcharhiniformes OR leucoraja OR pumpkinseed OR dosidicus OR acipenseriformes OR daubentonii OR emberizidae OR gadiformes OR hyraxes OR stizostedion OR wolverine OR wolverines OR lissotriton OR acanthurus OR centrarchidae OR gloydius OR laurasiatheria OR limosa OR psittacula OR leporidae OR proteidae OR zander OR zanders OR arapaima OR bagridae OR cyprinodontidae OR mithun OR pandion OR jackdaw OR jackdaws OR procyonidae OR carus OR jaculus OR salmoniformes OR "common sole" OR "common soles" OR protobothrops OR calamita OR brachyteles OR trionyx

OR turdidae OR boidae OR luscinia OR pugnax OR euarchontoglires OR saithe OR saithes OR symphalangus OR aardvark OR aardvarks OR oystercatcher OR oystercatchers OR arius OR corydoras OR poacher OR poachers OR aurochs OR cebuella OR crecca OR lemuridae OR sirenia OR lemmus OR perdix OR glires OR lepidosaur OR muskox OR deinagkistrodon OR pholidota OR holocephali OR cercopithecinae OR clariidae OR agapornis OR doryteuthis OR tyrannidae OR dicroglossidae OR godwit OR godwits OR monedula OR pongidae OR atheriniformes OR colobinae OR lophocebus OR atelidae OR cottidae OR leucopsis OR acanthuridae OR didelphimorphia OR elver OR elvers OR lapponica OR dermoptera OR "european hake" OR "european hakes" OR gerbillinae OR banteng OR hartebeest OR hartebeests OR hogget OR haematopus OR "anguis fragilis" OR "grey heron" OR "grey herons" OR "blue whiting" OR "blue whitings" OR furnariidae OR macrovipera OR esocidae OR

lapwing OR lapwings OR mylopharyngodon OR wallabia OR beloniformes OR potoroo OR potoroos OR "athene noctua" OR pleuronectidae OR bushbabies OR muscicapidae OR alligatoridae OR fuligula OR "bush baby" OR guineafowl OR spoonbill OR spoonbills OR viverridae OR catostomidae OR zebrafishes OR ibexes OR vendace OR estrildidae OR monotremata OR sepiella OR ambystomatidae OR shelduck OR shelducks OR treeshrew OR treeshrews OR hoplobatrachus OR pochard OR

hoolock OR hoolocks OR lynxes OR antilope OR antilopes OR blackbuck OR blackbucks OR cricetinae OR paramisgurnus OR skylark OR skylarks OR soleidae OR allobates OR "northern wheatear" OR "northern wheatears" OR pitheciidae OR takin OR theria OR vanellus OR galaxiidae OR lorisidae OR ostralegus OR palaeognathae OR "stone loach" OR alauda OR callitrichinae OR caniformia OR duttaphrynus OR ictaluridae OR osteoglossiformes OR poultries OR curema OR "ruddy turnstone" OR

"ruddy turnstones" OR sheatfish OR sunfishes OR centropomidae OR hemachatus OR platalea OR thamnophilidae OR "song thrush" OR atherinopsidae OR siluridae OR tadorna OR chroicocephalus OR ermine OR ermines OR gavialis OR ruffe OR tupaiidae OR diprotodontia OR hyaenidae OR antilopinae OR crocodylidae OR herpestidae OR hippopotamidae OR "northern shoveler" OR "round gobies" OR cheirogaleidae OR indriidae OR fundulidae OR pythonidae OR rhynchocephalia OR anodorhynchus OR "red-backed shrike" OR "red-backed shrikes" OR triakidae OR phalangeridae OR aoudad OR boreoeutheria OR "eurasian jay" OR "eurasian jays" OR feliformia OR haplorhini OR osteoglossidae OR paenungulata OR struthioniformes OR ferina OR sanderling OR sanderlings OR spheniscidae OR cuttlefishes OR cygnet OR dasycneme OR gadwall OR gadwalls OR "pelobates fuscus" OR wryneck OR wrynecks OR afrosoricida OR culaea OR "dover sole" OR "dover

soles" OR paralichthyidae OR passeridae OR osteolaemus OR "song thrushes" OR bluethroat OR bluethroats OR hydrophiidae OR megrim OR mephitidae OR strepsirhini OR tomistoma OR epidalea OR osmeriformes OR "bush babies" OR tarsiiform OR atelinae OR bufotes OR "eurasian coot" OR "eurasian coots" OR galagidae OR geopelia OR philomachus OR tubulidentata OR bombinatoridae OR pelobatidae OR tachysurus OR ailuridae OR woodlark OR woodlarks OR alcelaphinae OR redshank OR redshanks OR salientia OR "sand smelt" OR "sand smelts" OR woodmice OR woodmouse OR dasyproctidae OR "eurasian wigeon" OR "eurasian wigeons" OR garganey OR garganeys OR "lemon sole" OR "lemon soles" OR "common dab" OR "common dabs" OR graylag OR graylags OR leucorodia OR osphronemidae OR bewickii OR "common moorhen" OR "common moorhens" OR ecapodiformes OR gobbler OR gobblers OR odontophoridae OR paddlefishes OR salmonine OR esociformes OR "eurasian woodcock" OR "eurasian woodcocks" OR "european smelt" OR "european smelts" OR goldfishes OR tenches OR tyranni OR "common chaffinch" OR "common chaffinchs" OR "common redstart" OR "common redstarts" OR "common roach" OR "common roachs" OR "great knot" OR "great knots" OR potoroidae OR alytidae OR coregonine OR dipteral OR leveret OR "poeciliopsis gracilis" OR amphiumidae OR batrachoidiformes OR "bighead goby" OR heteropneustidae OR lullula OR "norway pout" OR "norway pouts" OR sipunculida OR dogfishes OR sebastidae OR tarsiidae

OR alethinophidia OR "common nase" OR "common nases" OR "common sandpiper" OR "common sandpipers" OR "eurasian blackcap" OR "eurasian blackcaps" OR pterocnemia OR syngnathiformes OR "common chaffinches" OR eupleridae OR octopodiformes OR phascolarctidae OR cophthalmidae OR "starry smooth-hound" OR "starry smooth-hounds" OR whitefishes OR cuniculidae OR "european sprat" OR "european sprats" OR "rosy bitterling" OR "rosy bitterlings" OR "common dace" OR "common daces" OR "lesser weever" OR "lesser weevers" OR scaldfish OR "water rail" OR "water rails" OR alouattinae OR centrarchiformes OR "common whitethroat" OR "common whitethroats" OR gavialidae OR "grey gurnard" OR "grey gurnards" OR lateolabracidae OR rheiformes OR "tub gurnard" OR "tub gurnards" OR "common chiffchaff" OR "common chiffchaffs" OR garfishes OR "lesser whitethroat" OR "lesser whitethroats" OR myoxidae OR seabasses OR spariformes OR umbridae OR "yellow boxfish" OR anabantiformes OR aotidae OR "common bleak" OR "common bleaks" OR "common rudd" OR "common rudds" OR "greater pipefish" OR hapale OR nandiniidae OR "stone loaches" OR whinchat OR whinchats OR acanthuriformes OR "brotula barbata" OR "common ling" OR "common lings" OR "common roaches" OR cottonrat OR cottonrats OR douroucoulis OR dromaiidae OR fitches OR fitchew OR galaxiiformes OR laprine OR saimiriinae OR solenette OR tarsii OR "tompot blenny" OR "common dragonet" OR "common dragonets" OR "longspined bullhead" OR "longspined bullheads" OR monotremate OR monotremates OR pempheriformes OR perdicinae

OR presbytini OR smegmamorpha OR "bighead gobies" OR "carangaria incertae sedis" OR coiidae OR "fivebeard rockling" OR foulmart OR foumart OR grasskeet OR "greater pipefishes" OR ibices OR millionfish OR muguliformes OR "norwegian topknot" OR peewit OR "red sea sailfin tang" OR rupicapras OR sheatfishes OR "tompot blennies" OR "twait shad" OR "yellow boxfishes")

# Supplementary material 5 - Report Expert Meeting

**Inhalation anaesthetics – deriving health-based safe exposure levels for workplace**

Date: Tue 7^th^ December 2021, 13:30-15:00 CET

Location: Teleconference

Participating experts

Prof. dr. Albert Dahan, anesthesiologist, Leiden University Medical Center, Leiden, The Netherlands

Dr. Sebastian Hoffmann PhD, ERT, seh consulting + services, Paderborn, Germany

Julia Menon MSc, systematic review specialist, France

Prof. dr. Merel Ritskes-Hoitinga PhD, evidence-based laboratory animal scientist, Radboudumc

Prof. dr. Gert Jan Scheffer MD PhD, anesthesiologist, Dept Anesthesiology, Radboudumc

Prof. dr. Vivi Schlünssen MD PhD, occupational physician, University of Aarhus, Denmark

NFU Workgroup Inhalation Anesthetics

Mrs. Anke Boumans-d’Onofrio MSc, Occupational and Environmental Service, Radboudumc, Nijmegen, The Netherlands

Mr. Hendrik Jan Jansen MSc, Amsterdam UMC, Amsterdam The Netherlands

Project team Radboudumc and Radboud University Nijmegen

Ms. Marije Buijs, student assistant

Dr. Carlijn Hooijmans PhD, systematic review specialist

Assoc. Prof. dr. Paul T.J. Scheepers PhD ERT RAH, toxicologist, occupational hygienist

Ms. Fréderique Struijs, student assistant

After a brief word of welcome the experts introduced themselves. This meeting is the first expert meeting to discuss how to establish a health-based recommended occupational exposure limit (OEL) for isoflurane and sevoflurane. Follow-up meetings will be planned in 2022 and 2023. This project should result in OELs for 8-hour (full shift) and 15-min (short-term exposure). This project builds on the experience from a similar effort to derive a recommendation for a health-based OEL for nitrous oxide that was completed in October 2021 (Menon et al., 2021). Most of the invited experts were also involved in the nitrous oxide project. The project team intends to publish the systematic review protocol and the full systematic review including the OEL recommendation for both substances. Experts can be involved as co-authors.

The experts have an interest in the motivation for the selection for isoflurane and sevoflurane and not desflurane. This selection is based on the current use of inhalation anesthetics by the academic hospitals who collaborate in the Netherlands Federation of Academic Hospitals (NFU). Sevoflurane is much used in these hospitals. Isoflurane is nowadays mainly used in animal research facilities. Desflurane was also considered but is not much used in the Netherlands because of its risk in some procedures and also for technical and marketing reasons. From a wider (global) perspective it would be interesting to also consider desflurane for a future project.

The current OEL recommendations for isoflurane and sevoflurane will be based on the following steps: (a) a systematic search and selection and appraisal of the quality of available evidence, (b) dose-response modelling of animal data, and (c) extrapolation to humans for two reference time-frames (provided that data are available to support this). Animal data will be used because of limitations of the available human evidence. In occupational settings, inhalation anesthetics has been associated with adverse reproductive and developmental outcomes, mainly spontaneous miscarriages over many decades (see Boivin et al., 1997 for an overview). A small study by Peelen et al. (1999) that was carried out in the Netherlands reported on an increased frequency of malformations at birth and spontaneous abortions associated with working as a nurse in an operation room or assisting in tonsillectomies. These observations were statistically significant, also after adjustment for other known risk factors for reproductive health. In a recent study performed in nurses in Canada the outcome of more than 1000 pregnancies with congenital malformations were associated with exposure to exposure to inhalation anesthetics (combined exposure including isoflurane and sevoflurane). In this study malformations were reported to be statistical significantly increased but these results were not adjusted for relevant co-exposures other than inhalation anesthetics.

The Nordic expert group evaluated available evidence in 2009 and concluded that human studies cannot be used to derive an OEL because of the lack of studies on long-term exposure in a low concentration range. According to the expert group, isoflurane was the most studied anesthetic in single exposures. Sevoflurane was studied to a much more limited extent and for desflurane the available human data was considered ‘very poor’ in the 2009 evaluation. Previous evaluations for nitrous oxide have also considered human evidence as insufficient for deriving an OEL. Therefore, in this project we will focus on the available animal studies. The experts indicated that it will be challenging to extrapolate from animal studies to humans. It was also clarified that the study by Peelen was not considered in the evaluation by the Nordic Expert Group as it was only available a as report by the Ministry of Social Affairs and Employment (in Dutch) and not published in a peer-reviewed English literature.

The main aim of the meeting was to discuss the draft protocols for the systematic reviews for isoflurane and sevoflurane. The two protocols for isoflurane and sevoflurane were described separately according to a standard format developed by SYRCLE and were provided to the participants two weeks in advance of the meeting.

The content of the protocols was introduced and motivated. The main questions related to the protocols were related to the proposal by the project team to use a cut-off for the exposure level of 1 % by volume and include studies with an exposure lower than this value. The justification for that is that studies with higher concentrations likely relate to research questions from a clinical perspective whereas studies with much lower concentrations are considered more relevant to workplace exposures. This proposed cut-off of 1 % was previously used in the study on nitrous oxide.

The project team proposes to limit the effort for risk bias assessment to the subset selected for dose-response modelling on a specific outcome agreed upon with the experts similar to the nitrous oxide case. The experts understand this strategy but would be interested to have at least some insight in the overall quality of the evidence. From previous assessment of the risk of bias the project team expects rather low risk of bias for most if not all animal studies. The project team agrees that it is important to justify the choice for the subset of studies used for the dose-response modelling based on study characteristics and study quality. Possibly the quality assessment will be enlarged, the project team will discuss this further. Both study protocols will be registered online at PROSPERO and the project team plans to submit to separate SR protocol manuscripts to an international peer-review journal.

The preliminary search strategy and some early results from the search were presented. The search strategy was developed with input from a librarian. Searches were conducted in Pubmed, Embase and Web of Science. The search resulted in high numbers of retrieved studies. Because of the high number of studies retrieved, machine learning using EPPI-Reviewer (see <https://eppi.ioe.ac.uk/>) will be used to support the screening and selection. To determine an optimized machine learning algorithm a set of 1000 to 3000 title-abstracts will be screened initially by two reviewers independently using Rayyan (see <https://www.rayyan.ai/>). Conflicts will be reconciled by a third reviewer. For the machine learning the performance target is a sensitivity of 95% and specificity of 80-85%. Because of the very large number of retrieved studies snow balling approaches will not be considered.

References

Boivin, J.F., Risk of spontaneous abortion in women occupationally exposed to anaesthetic gases: a meta-analysis. Occup Environ Med, 1997. 54(8): p. 541-8.

Health Council of the Netherlands: Committee for Compounds toxic to reproduction. Isoflurane; Evaluation of the effects on reproduction, recommendation for classification. The Hague: Health Council of the Netherlands, 2002; publication no. 2002/13OSH.

Nordic Expert Group (2009) The Nordic Expert Group for Criteria Documentation of Health Risks from Chemicals 141. Isoflurane, sevoflurane and desflurane. Nr 2009;43(9).

Peelen, S., et al., Reproductie-toxische eﬀecten bij ziekenhuispersoneel. . 1999, Den Haag: Elsevier Bedrijfsinformatie BV.
